# RSV-specific airway resident memory CD8+ T cells and differential disease severity after experimental human infection

**DOI:** 10.1038/ncomms10224

**Published:** 2015-12-21

**Authors:** Agnieszka Jozwik, Maximillian S. Habibi, Allan Paras, Jie Zhu, Aleks Guvenel, Jaideep Dhariwal, Mark Almond, Ernie H. C. Wong, Annemarie Sykes, Matthew Maybeno, Jerico Del Rosario, Maria-Belen Trujillo-Torralbo, Patrick Mallia, John Sidney, Bjoern Peters, Onn Min Kon, Alessandro Sette, Sebastian L. Johnston, Peter J. Openshaw, Christopher Chiu

**Affiliations:** 1 National Heart and Lung Institute, Imperial College London, London W2 1PG, UK; 2 Centre for Infectious Disease, Division of Vaccine Discovery, La Jolla Institute of Allergy and Immunology, 9420 Athena Circle, La Jolla, California 92037, USA

## Abstract

In animal models, resident memory CD8+ T (Trm) cells assist in respiratory virus elimination but their importance in man has not been determined. Here, using experimental human respiratory syncytial virus (RSV) infection, we investigate systemic and local virus-specific CD8+ T-cell responses in adult volunteers. Having defined the immunodominance hierarchy, we analyse phenotype and function longitudinally in blood and by serial bronchoscopy. Despite rapid clinical recovery, we note surprisingly extensive lower airway inflammation with persistent viral antigen and cellular infiltrates. Pulmonary virus-specific CD8+ T cells display a CD69+CD103+ Trm phenotype and accumulate to strikingly high frequencies into convalescence without continued proliferation. While these have a more highly differentiated phenotype, they express fewer cytotoxicity markers than in blood. Nevertheless, their abundance before infection correlates with reduced symptoms and viral load, implying that CD8+ Trm cells in the human lung can confer protection against severe respiratory viral disease when humoral immunity is overcome.

## Abstract (web summary)

Respiratory syncytial virus (RSV) is a common cause of respiratory tract infections. Here the authors analyse cellular immune responses of individuals experimentally infected with RSV and reveal the presence of high frequencies of virus- specific resident memory CD8^+^ T cells in the airway, which correlate with improved viral control.

## Graphical abstract

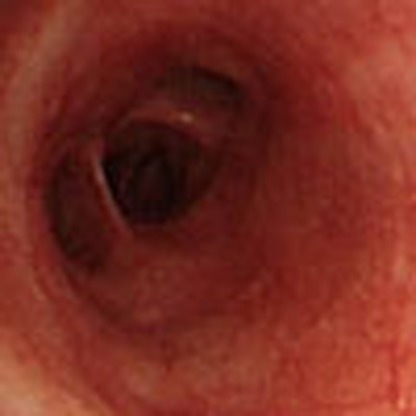

CD8+ T cells are essential effectors that eliminate intracellular pathogens and confer protection against symptomatic reinfection via immune memory^1^. In animal models of respiratory virus infections such as respiratory syncytial virus (RSV) and influenza, memory CD8+ T cells reduce viral replication, prevent infection or decrease disease severity, and confer cross-protection against antigenically distinct strains^2^. However, while some vaccine candidates against RSV and influenza may have the capacity to induce CD8+ T cells, they have not yet been shown clinically to improve protection^3^,^4^,^5^.

RSV is globally the commonest cause of lower respiratory tract infection in children, leading to an estimated 3.4 million hospitalizations each year^6^. It is also a major contributor to mortality in older and immunosuppressed adults^7^. Recurrent symptomatic RSV infection occurs throughout life even with a healthy immune system and limited viral antigenic variation^8^. Therefore, characterizing immune responses required for robust protection has been problematic and effective vaccines remain a major clinical need^2^. We recently showed that anti-RSV IgA in the nasal mucosa correlated strongly with protection from infection, but that the high levels required for immunity are poorly maintained, allowing recurrent infection^9^. Despite this, most older children and young adults suffer only minor symptoms, implying that when antibodies fail to prevent infection, cell-mediated immunity reduces disease severity.

In mice, depletion of RSV-specific CD8+ T cells leads to prolonged viral replication, while adoptive transfer of virus-specific memory cells enhances virus clearance^10^,^11^. However, the absence of T cells also leads to reduced symptom severity and transfer of RSV-specific memory T cells worsens disease, indicating that harmful immunopathology can outweigh the benefits of cell-mediated viral clearance under certain circumstances^12^,^13^. In humans, the role of CD8+ T cells remains less clear with evidence of their protective role mainly limited to observations of children with T-cell defects (who suffer more severe disease with prolonged viral shedding)^14^. In influenza, correlations between memory T cells in the blood and reduced severity of disease on subsequent infection have been shown^15^,^16^, but no such evidence exists in RSV and the extent to which T cells contribute to protection or pathology in this and other respiratory viral infections remains unknown.

Respiratory viruses are usually confined to the lung with systemic spread only in the worst cases^17^. Virus-specific CD8+ T cells in peripheral blood are therefore unlikely in most situations to be directly relevant to protection. Instead, studies of a range of tissues have recently defined a subset of non-circulating memory T cells specialized to protect sites of pathogen entry^18^. These resident memory T (Trm) cells are not only poised for rapid killing on virus re-encounter but may also exhibit innate-like sensing functions^19^. In mouse models of influenza, CD4+ and CD8+ Trm cells in the lung confer greater protection than spleen-derived cells^20^,^21^. However, restrictions on sampling of human lungs mean that little is known about these Trm cells except that they are abundant in non-inflamed lung from tumour excisions or donated organ tissue^22^,^23^.

We investigated the CD8+ T-cell response to experimental RSV infection in 49 healthy adult volunteers, around half of who also underwent serial bronchoscopy. While controlled for variations in viral inoculum and co-morbidities, this cohort nevertheless represented a genotypically diverse antigen-experienced population that allowed characterization of the breadth of virus-specific CD8+ T-cell responses and identification of novel immunodominant and subdominant epitopes. Analysis using major histocompatibility complex (MHC)-peptide tetramers revealed highly contrasted kinetics, phenotypes and functionality of RSV-specific CD8+ T cells in the lower respiratory tract compared with blood, the diversity of which allowed us to infer a specialised role in protection against RSV disease.

## Results

### Experimental RSV infection causes upper tract disease

**Figure 1:**
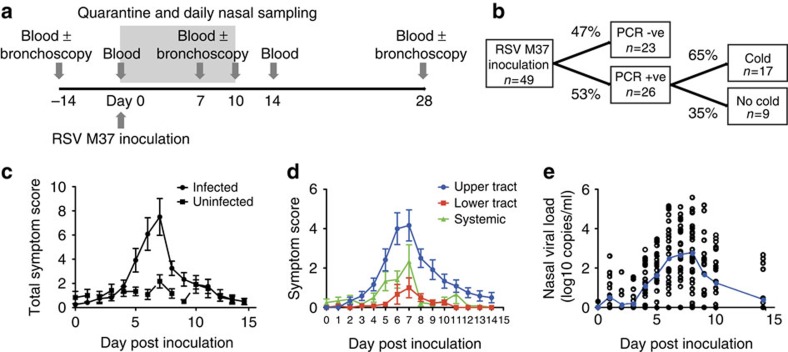
Inoculation of healthy adults with RSV causes upper respiratory tract infection. Forty-nine healthy adult volunteers were inoculated with RSV Memphis 37. (**a**) The study design is shown. (**b**) Clinical outcomes are shown. (**c**) Self-reported symptoms scores of 49 subjects were recorded daily. Cumulative data are shown as mean±s.e.m. Infected individuals were defined by having had RSV detected from nasal lavage on at least 2 consecutive days by qPCR of nasal lavage. (**d**) Symptoms from 49 subjects were categorized as originating from the upper respiratory tract (blue), lower respiratory tract (red) or as systemic (green) according to previously described criteria (see Methods). Data are shown as mean± s.e.m. (**e**) Viral load of 49 individuals was determined by *N* gene qPCR from nasal lavage. Individual data points and means are shown.

We enrolled 49 healthy adults aged 18–50 years (median 20.5 years; Supplementary Table 1). Two weeks and immediately before inoculation, they underwent blood and nasal sampling (Fig. 1a). All individuals were then inoculated with 10^4^ plaque-forming units of RSV A Memphis 37 (RSV M37) by intranasal drops as previously described^9^, followed by sequential blood and nasal samples up to 6 months later.

Twenty-six subjects (53%) developed PCR+ infection (Fig. 1b). Of these, 17 (65%) suffered a ‘common cold' according to standardized criteria (see Methods), while 9 (35%) reported minimal or no symptoms. Symptoms in infected individuals peaked around day 7 post inoculation (Fig. 1c). While almost all symptomatic individuals complained of upper respiratory symptoms, lower respiratory tract and systemic symptoms were unusual (Fig. 1d). In infected individuals, viral load peaked at days 7–8 post infection with a mean of 2.76 log_10_ copies per ml (±s.e.m. 0.322; Fig. 1e), correlating with self-reported symptoms (Supplementary Fig. 1).

### RSV causes inflammation in the lower respiratory tract

**Figure 2:**
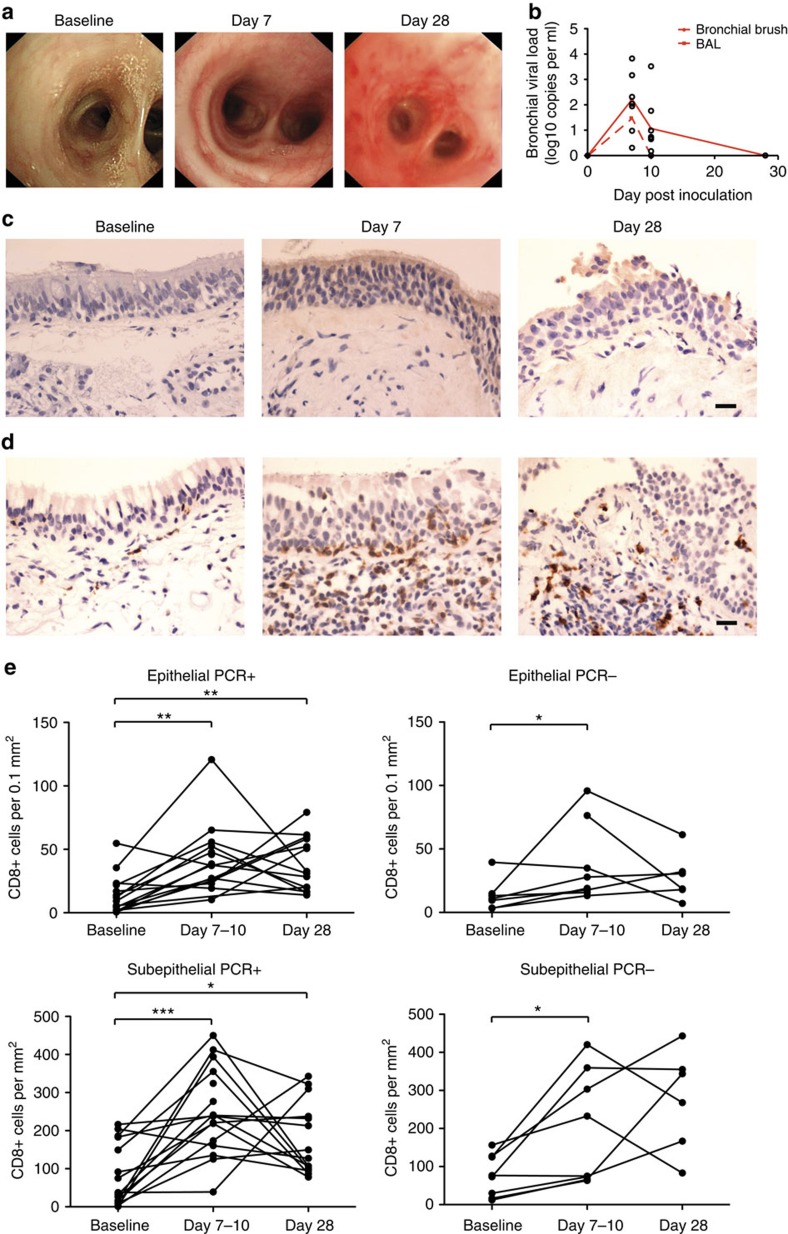
RSV infection of healthy adults causes widespread lower respiratory tract inflammation. Twenty-four volunteers underwent serial bronchoscopic procedures 14 days before inoculation with RSV M37, 7 or 10 days and 28 days post inoculation. (**a**) The macroscopic appearance of the airways was noted by bronchoscopists blinded to the infection status of the subject. One representative subject is shown. (**b**) Viral load was determined from bronchial brushings (solid line) and bronchoalveolar lavage (dashed line) by qPCR. Cumulative data are shown as mean±s.e.m. (**c**) RSV antigen (brown) was detected in bronchial biopsies by immunohistochemistry. One representative donor is shown. (**d**) CD8+ cells (brown) were identified in bronchial biopsies by immunohistochemistry. Scale bar, 20 μm. (**e**) CD8+ cells were enumerated in bronchial biopsies of infected individuals (with RSV detected by qPCR in bronchial brushings) and PCR− individuals. Data are presented as number of positive cells per square millimetre of subepithelium or per 0.1 mm^2^ of epithelium. Significant *P* values for two-tailed Wilcoxon matched pairs tests are shown (*P<0.05, **P<0.01, ***P<0.001) comparing (with baseline) epithelial PCR+ samples at day 7–10 (*P*=0.0012) and day 28 (*P*=0.004), epithelial PCR− samples at day 7–10 (*P*=0.0469), subepithelial PCR+ samples at day 7–10 (*P*=0.0004) and day 28 (*P*=0.0419), and subepithelial PCR− samples at day 7–10 (*P*=0.0156).

Twenty-four volunteers additionally underwent serial bronchoscopy (Supplementary Table 2). Thus, endobronchial biopsies, bronchial brushings and bronchoalveolar lavage (BAL) were obtained 14 days before inoculation and 7 or 10 and 28 days after. Of these, 50% developed RSV infection. In view of the predominantly upper respiratory disease, we were surprised to find strikingly extensive macroscopic inflammation involving the lower respiratory tract of infected individuals (Fig. 2a; Supplementary Table 3). Lower airways involvement was supported by quantitative PCR (qPCR) of bronchial brushings, which detected RSV in all day 7 samples and 4/5 infected subjects at day 10, again peaking around 7 days (mean 2.22 log_10_ ml^−1^±s.e.m. 0.416; Fig. 2b). RSV could also be measured in BAL in 5/7 individuals at day 7 at lower copy numbers (peak 1.48 log_10_ ml^−1^±s.e.m. 0.412). Interestingly, virus was detected in bronchial brushings of an additional four subjects in whom no virus was found in nasal lavage. These were found at low-to-moderate copy numbers (0.31 log_10_ ml^−1^ at day 7, and 0.69, 0.17 and 1.76 log_10_ ml^−1^ at day 10) and were reproducible on repeat assay.

To confirm the unexpected presence of RSV in the lower airway, endobronchial biopsies were analysed by immunohistochemistry (Fig. 2c). While RSV antigen was not found pre-inoculation, all individuals with PCR-detectable virus in the nose had at least moderate antigen staining at both acute and convalescent time points. Of these, 3/11 had extensive RSV antigen in bronchial biopsies at day 7–10 and 3/10 at day 28 (Supplementary Table 4). In contrast, PCR− subjects had, at most, staining of isolated epithelial cells, suggesting the presence of RSV antigen but no onward spread. RSV antigen was associated with infiltration of CD8+ T cells (Fig. 2d). In those with PCR+ bronchial brushings, CD8+ T cells increased significantly in both the epithelial and subepithelial layers at acute and convalescent time points (Fig. 2e). In contrast, while CD8+ T cells did increase transiently in the biopsies of PCR− individuals, these were less consistently seen and did not remain significantly elevated at day 28. Thus, experimental infection of adults with RSV can lead to virus replication in the lower airway, which causes marked inflammation associated with infiltration of CD8+ T cells.

### RSV viral load drives CD8+ T-cell proliferation

**Figure 3:**
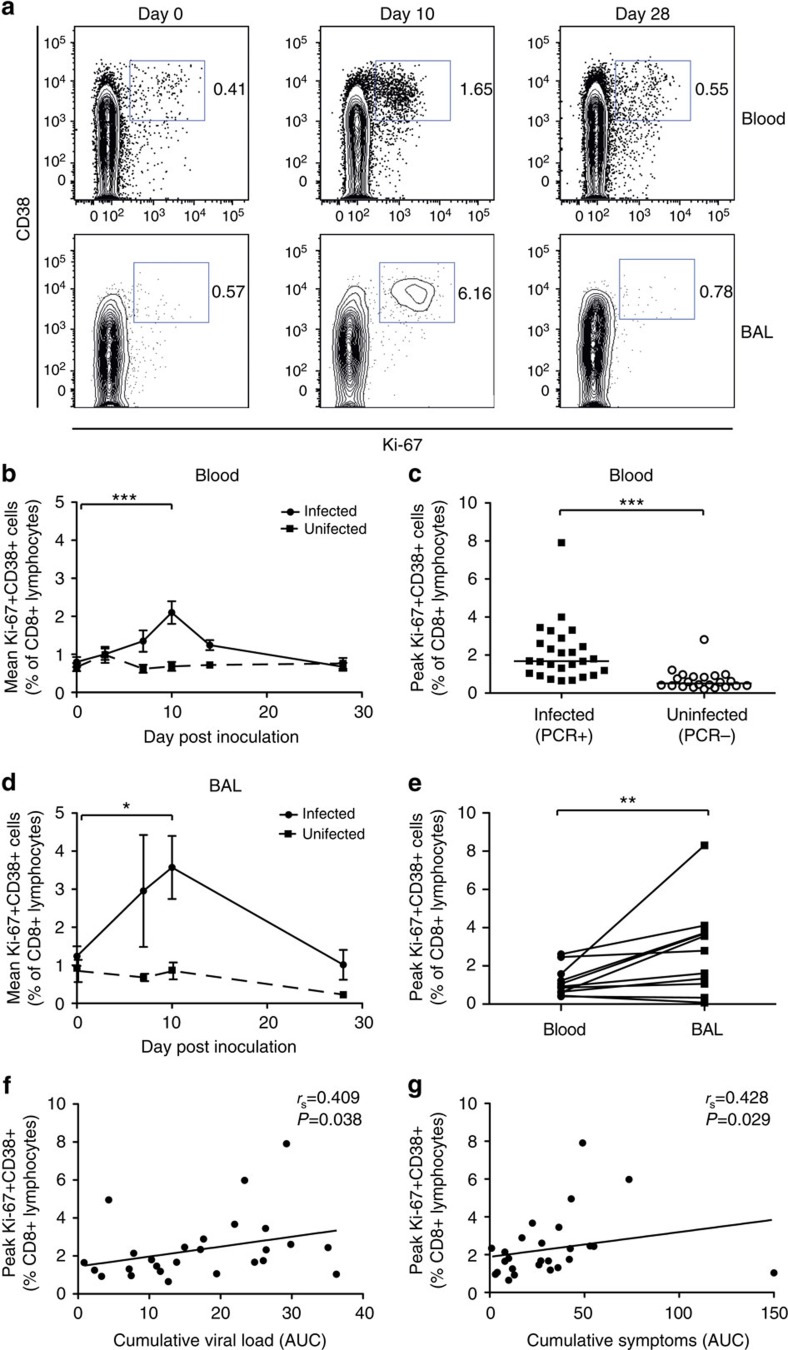
RSV infection induces short-lived activation and proliferation of CD8+ T cells in peripheral blood. Forty-nine adult volunteers were inoculated with RSV Memphis 37, serial blood (*n*=49) and BAL (*n*=24) samples were stained with anti-CD3, CD8, Ki-67 and CD38, and analysed by flow cytometry. (**a**) Plots are gated on CD3+CD8+ lymphocytes. Numbers represent percentage of CD8+ T cells. One representative subject is shown. (**b**) Mean and s.e.m. of Ki-67+CD38+ CD8+ T-cell frequencies in the blood of infected (PCR+) and challenged but uninfected (PCR−) are shown. The *P* value for a two-tailed Wilcoxon matched pairs test is shown (***P<0.001). (**c**) Frequencies of Ki-67+CD38+ cells as a proportion of CD8+ T lymphocytes were determined at day 10 post inoculation in infected and uninfected individuals. Mean±s.e.m. are shown with the *P* -value for a two-tailed Mann–Whitney test (***P=0.001). (**d**) Mean and s.e.m. of Ki-67+CD38+ CD8+ T-cell frequencies in the BAL of infected (PCR+) and challenged but uninfected (PCR−) are shown. The significant *P* value for a two-tailed Wilcoxon matched pairs test is shown (*P=0.0317). (**e**) Frequencies of Ki-67+CD38+ cells, as a proportion of CD8+ T lymphocytes were determined at day 10 post inoculation in matched blood and BAL samples. The significant *P* value for the Wilcoxon matched pairs tests is shown (**P=0.0059). Correlations between peak frequencies of Ki-67+CD38+ CD8+ T cells 10 days post infection in blood and (**f**) cumulative viral load and (**g**) symptoms (trapezoidal area under the curve (AUC)) are shown using non-linear regression and Spearman's rank correlation.

To further investigate the kinetics of the response, CD8+ T cells induced by RSV were tracked by flow cytometry using markers of proliferation (Ki-67) and activation (CD38; Fig. 3a). From day 7 post infection, activated CD8+ T cells in blood expanded peaking around day 10 (mean 2.1%±s.e.m. 0.297 of CD8+ lymphocytes), after which Ki-67+CD38+ CD8+ T cells rapidly returned to baseline frequencies (Fig. 3b). No significant proliferative response was seen in PCR− individuals, indicating that antigen challenge without viral replication was not sufficient to induce CD8+ T-cell activation (Fig. 3c). Similarly, frequencies of activated CD8+ T cells increased in BAL (Fig. 3d). However, the average frequency was significantly higher than in blood (*P*=0.0098 Wilcoxon matched pairs test; Fig. 3e). The appearance of activated CD8+ T cells coincided with the fall in viral load (Fig. 1e). Antigen availability and inflammation both contribute to T-cell activation, and indeed, cumulative viral load and symptoms (by trapezoidal area under the curve) both correlated with the peak frequency of activated CD8+ T cells (Fig. 3f,g), suggesting that increased virus burden and/or inflammatory changes in the airway drive T-cell activation, which in turn leads to viral clearance.

### HLA-associated immunodominance hierarchies against RSV

**Figure 4:**
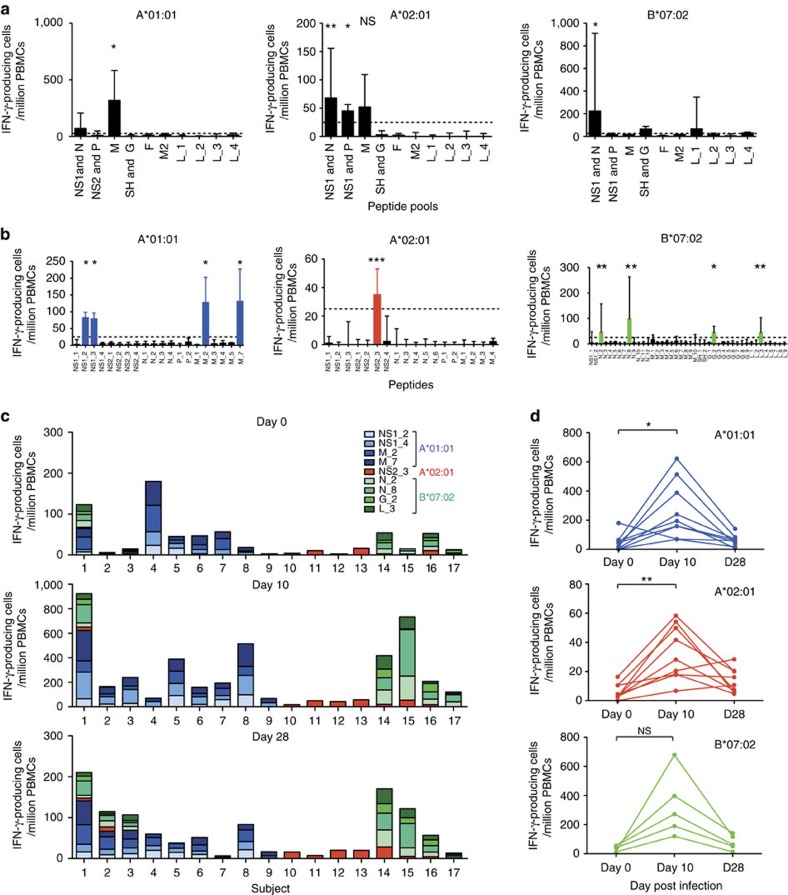
Immunodominance hierarchies in MHC class-I-restricted RSV epitope-specific CD8+ T-cell responses. Epitopes were predicted *in silico* for binding and used to stimulate day 10 PBMCs from HLA-matched infected volunteers with sufficient cells (HLA A*01:01, *n*=4; A*02:01, *n*=5; and B*07:02, *n*=4) in interferon-γ (IFN-γ) ELISpot assays. (**a**) Peptides were pooled according to their originating RSV protein. Two-tailed Mann–Whitney tests were carried out between peptide pools and negative control wells (A*01:01_NS1/N, *P*=0.0286; A*02:01_NS1/N, *P*=0.0079; A*02:01_NS2/P, *P*=0.0119; and B*07:02_NS1/N, *P*=0.0286). (**b**) Individual peptides from positive pools were subsequently tested separately. Dotted lines represent a cutoff of 25 spots per 10^6^ PBMCs. Means±s.e.m. and *P* values for unpaired Mann–Whitney tests are shown (A*01:01_NS1_2, *P*=0.0286; A*01:01_NS1_4, *P*=0.0286; A*01:01_M_2, *P*=0.0294; A*01:01_M_7, *P*=0.0294; A*02:02_NS2_3, *P*=0.0002; B*07:02_N_2, *P*=0.0079; B*07:02_N_8, *P*=0.0079; B*07:02_G_2, *P*=0.0278; and B*07:02_L_3, *P*=0.0079). (**c**) The median frequencies of IFN-γ+ cells in response to individual RSV epitopes presented by HLA-A*01:01 (blues), A*02:01 (reds) and B*07:02 (greens) are shown in 17 HLA-matched RSV-infected individuals at day 0, 10 and 28 post infection. (**d**) The frequencies of median total IFN-γ-producing cells at baseline, day 10 and day 28 post infection in response to HLA-A*01:01 (blue)-, A*02:01 (red)- and B*07:02 (green)-restricted epitopes in 17 HLA-matched infected individuals are shown. *P* values for Wilcoxon matched pairs tests are shown (A*01:01, *P*=0.0117; A*02:01, *P*=0.0039; and B*07:02, *P*=0.125; NS (not significant)=*P*>0.05, *P<0.05, **P<0.01, ***P<0.001).

Antigenic targets of RSV-specific CD8+ T cells have not been systematically investigated and only a few isolated epitopes have been identified^24^,^25^. To define the breadth of these responses, we generated a peptide library from RSV M37 predicted to bind the most common human leukocyte antigen (HLA)-A and HLA-B alleles using the Immune Epitope Database consensus prediction tools^26^. The top 1% of predicted MHC-binding peptides for each RSV protein was pooled and peptide pools used to screen peripheral blood mononuclear cells (PBMCs) from subjects at day 10 post infection by interferon-γ (IFN-γ) enzyme-linked immunospot (ELISpot). Pools containing peptides from M, N, P, NS1 and NS2 (all internal proteins) induced IFN-γ production (Fig. 4a).

Pools that stimulated responses were subsequently deconvoluted to individual epitopes (Fig. 4b; Supplementary Table 5). In the context of A*01:01, two epitopes (previously described YLEKESIYY [YLE]^25^ and the novel VTDNKGAFKY [VTD]) that elicited dominant responses were identified in the M protein and one subdominant epitope (LSDSTMTNY [LSD]) in the NS1 protein (Supplementary Table 5). A 10-mer differing from LSD in one additional amino acid also induced responses but had a 100-fold lower MHC-binding affinity. YLE and VTD were conserved with the prototypic RSV A2 strain. Only a single A*02:01-restricted epitope (FLVNYEMKL [FLV]) was identified, derived from the NS2 and conserved with RSV A2. Finally, with B*07:02, four epitopes were defined: NPKASLLSL (NPK, described previously^25^) and QVMLRWGVL (QVM) from N protein (previously shown as part of a longer polypeptide to induce IFN-γ in some individuals^27^); IPAYRTTNY (IPA) from L; and KPNIRTTLL (KPN) from G (not conserved with RSV A2). VTD, LSD and FLV have previously been predicted but not confirmed as epitopes on the basis of HLA-binding affinity alone^28^. Indeed, all but three epitopes exhibited moderate to good measured MHC peptide-binding affinity (<500 nM). Importantly, each HLA allele presented epitopes from different RSV proteins with no overlap in the specificities of CD8+ T cells targeted against them.

In subjects with sufficient PBMCs, RSV-specific CD8+ T-cell responses were analysed before, during and after infection using IFN-γ ELISpot. Before infection, epitope-specific cells were rare, with a median (interquartile range (IQR)) of 45 (6–61) spot-forming units (SFU) per million PBMCs (A*01:01); 28 (13–52) SFU per million PBMCs (B*07:02); and <5 (<5–11) SFU per million PBMCs (A*02:01; Fig. 4c,d; Supplementary Table 6). At the peak of the CD8+ T-cell response, these had increased by ∼10-fold to 194 (114–452) SFU per million PBMCs for A*01:01; 231 (138–577) SFU per million PBMCs for B*07:02; and 28 (18–52) SFU per million PBMCs for A*02:01. The extent of expansion between the three HLA-restricted responses was similar (Fig. 4d; Supplementary Fig. 2). By day 28, these populations had contracted but remained enlarged compared with baseline in most individuals.

Throughout the course of infection, the immunodominance hierarchy was maintained both between individuals and within subjects who expressed two or three of these HLA alleles. Thus, A*01:01-restricted responses were consistently greater than those against B*07:02-restricted epitopes and those against the A*02:01-restricted FLV epitope were ∼10-fold lower. However, the FLV response was more prominent in individuals without the A*01:01 or B*07:02 alleles, suggesting the effect of immunodomination. Thus, we identified several new RSV epitopes (both immunodominant and subdominant) for three common HLA alleles with potential as vaccine targets. The immunodominance hierarchies indicated marked quantitative differences in responses determined by HLA type and the influence of HLA alleles on each other when co-expressed.

### RSV-specific CD8+ T cells accumulate in convalescent airways

**Figure 5:**
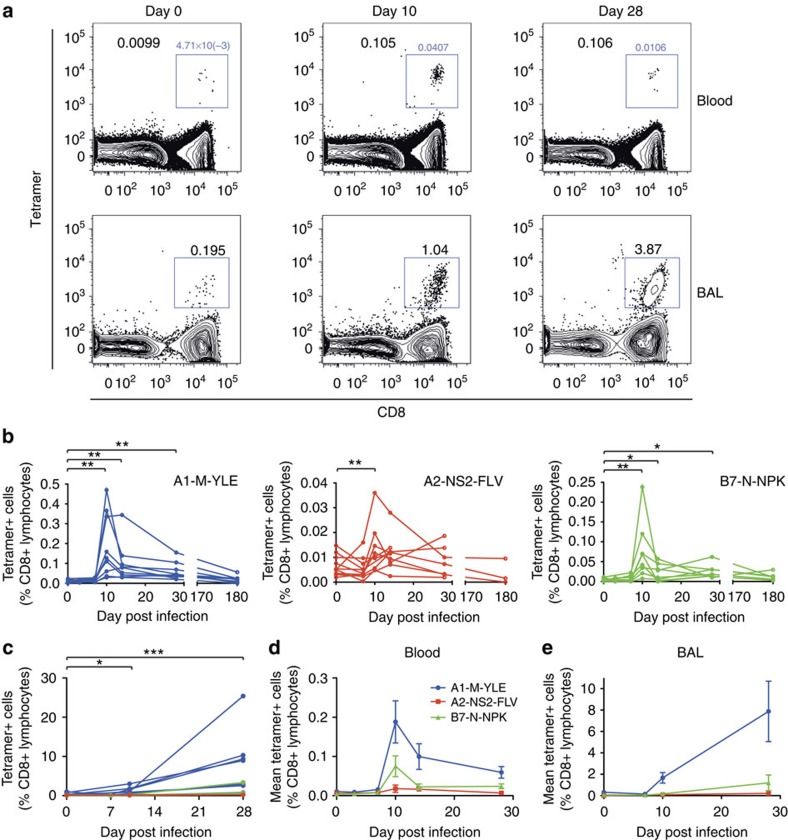
RSV antigen-specific CD8+ T-cell kinetics diverge in BAL compared with blood. Whole blood/PBMCs and BAL from RSV-infected individuals were co-stained with anti-CD3, CD8 and tetramers, and then analysed by flow cytometry. (**a**) Numbers represent the percentage of A1-M-YLE+ CD8+ T cells as a proportion of CD3+ lymphocytes at day 0, 7, 10, 14 and 28 post inoculation. Representative plots for one subject gated on CD3+ lymphocytes are shown. (**b**) The frequencies of A1-M-YLE (*n*=9), A2-NS2-FLV (*n*=10) and B7-N-NPK (*n*=8) tetramer-positive cells in blood as a proportion of CD8+ T cells are shown up to 6 months follow-up. *P* values for Wilcoxon matched pairs tests compared with day 0 are shown (A1-M-YLE day 10, *P*=0.0039; day 14, *P*=0.0039; day 28, *P*=0.0039; A2-NS2-FLV day 10, *P*=0.0488; B7-N-NPK day 10, *P*=0.0078; day 14, *P*=0.0223; and day 28, *P*=0.0207). (**c**) The frequencies of A1-YLE, A2-NS2-FLV and B7-N-NPK tetramer-positive cells in BAL as a proportion of CD8+ T cells are shown. *P* values for Wilcoxon matched pairs tests are shown comparing day 10 (*P*=0.0156) and day 28 (*P*=0.0002) frequencies with baseline. Abbreviated *P* values are shown: NS (not significant)=*P*>0.05, *P<0.05, **P<0.01. Mean±s.e.m. of epitope-specific CD8+ T-cell responses are shown in (**d**) blood (*n*=20) and (**e**) BAL (*n*=13).

MHC peptide tetramers were constructed using three immunodominant epitopes (A1-M-YLE, A2-NS2-FLV and B7-N-NPK) to investigate RSV-specific CD8+ T cells in further detail by flow cytometry in blood and BAL (Fig. 5a). In blood at baseline, tetramer+ cells were found at low frequencies ranging from undetectable to 0.03% of CD8+ lymphocytes (Fig. 5b,d). After day 7 post infection, these expanded coincident with the fall in viral load (Fig. 1e). They peaked at day 10 post infection with up to a mean 18-fold increase, after which epitope-specific populations contracted rapidly. While tetramer+ cells still remained elevated in most individuals 28 days post infection, their numbers were not maintained long term, so that by 6 months post infection there was no statistical difference compared with their preinfection levels.

Analysis of matched BAL samples from the same infected subjects, however, revealed an unexpected pattern of CD8+ T-cell kinetics that diverged markedly from those in blood (Fig. 5c). While in this compartment, tetramer+ cells also started increasing in frequency after 7 days, at both day 10 and day 28 they were significantly more frequent than in blood (*P*=0.0078 and 0.0002, respectively, Wilcoxon matched pairs test), with a sevenfold higher mean frequency of A1-M-YLE+ cells in BAL than in blood at day 10 (Fig. 5d,e). Furthermore, the frequency of tetramer+ cells continued to rise in BAL into the convalescent period despite the absence of symptoms or virus detection by qPCR, while contraction of these populations had occurred in blood. Therefore, at day 28 post infection, the mean frequency of A1-M-YLE+ cells was 114-fold greater in BAL than in blood.

### Phenotypically distinct RSV-specific CD8+ T cells in airways

**Figure 6:**
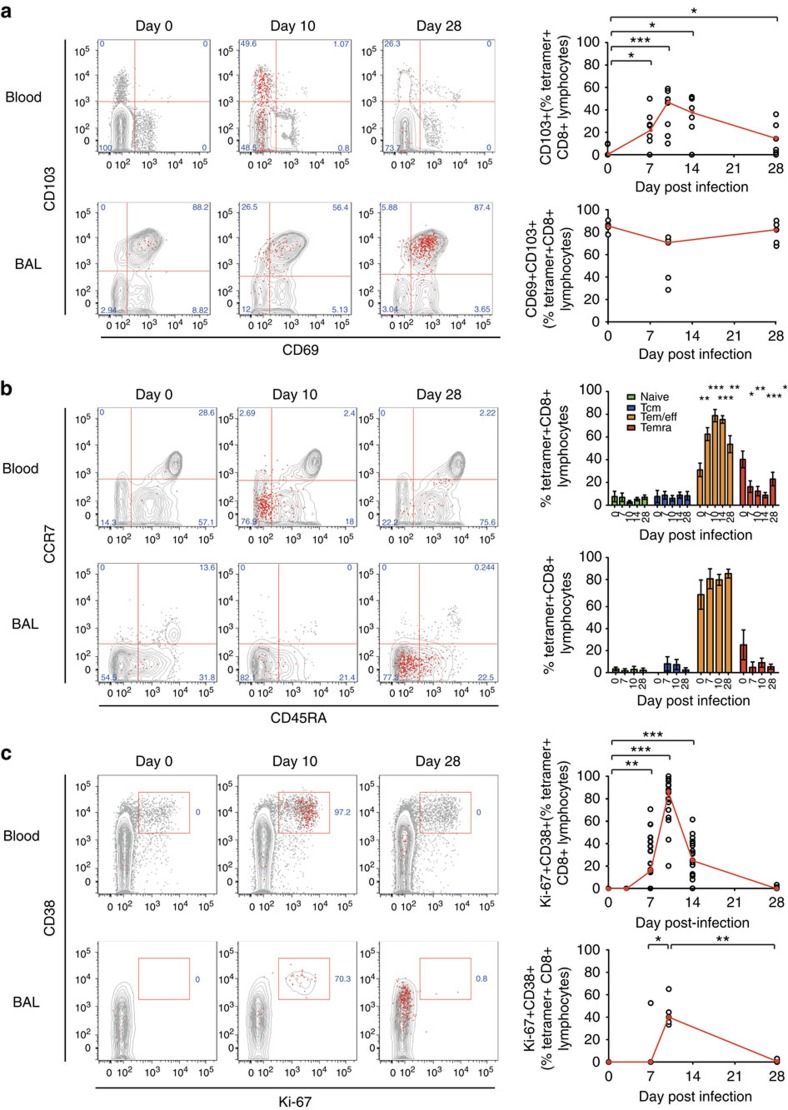
RSV-specific CD8+ T cells in BAL display a distinctive resident memory phenotype. Tetramer+ CD8+ T cells in blood and BAL were co-stained for markers to assess their differentiation status. (**a**) CD69 and CD103 as canonical markers of resident memory CD8+ T cells are shown in blood (*n*=9) and BAL (*n*=5) from infected volunteers. Significant *P* values for two-tailed Wilcoxon matched pairs tests in blood compared with baseline are shown (day 7, *P*=0.0313; day 10, *P*=0.0039; day 14, *P*=0.0313; and day 28, *P*=0.0313). (**b**) Memory markers CD45RA and CCR7 are shown in blood (*n*=19) and BAL (*n*=8). Mean±significant *P* values for two-tailed Wilcoxon matched pairs tests compared with baseline are shown in blood for T-effector/effector memory cells (day 7, *P*=0.0034; day 10, *P*=0.0002; day 14, *P*=0.0002; day 28, *P*=0.0067) and effector memory T cells re-expressing CD45RA (day 7, *P*=0.0443; day 10, *P*=0.0025; day 14, *P*=0.0003; day 28, *P*=0.0135). (**c**) Proliferation and activation markers Ki-67 and CD38 are shown in blood (*n*=19) and BAL (*n*=8). Significant *P* values for two-tailed Wilcoxon matched pairs tests are shown compared with baseline in blood (day 7, *P*=0.0025; day 10, *P*=0.0001; and day 14, *P*=0.0005) and BAL (day 7 versus day 10, *P*=0.0444; and day 10 versus day 28, *P*=0.0022 as no Ki-67+CD38+ cells were found in any baseline samples). Throughout, representative plots from a single subject at day 0, 10 and 28 post infection are shown with tetramer+ cells as red dots and total CD8+ T cells in grey contours. Summarized data are shown for all RSV-infected subjects (who could be analysed using tetramers) with median frequencies shown in red. Abbreviated *P* values are shown: NS (not significant)=*P*>0.05, *P<0.05, **P<0.01.

In view of the strikingly divergent kinetics in BAL and blood, we went on to analyse the phenotypic differences between RSV-specific CD8+ T cells in the two compartments. In BAL at rest, almost all (80–90%) tetramer+ cells displayed the canonical CD69+CD103+ Trm phenotype (Fig. 6a). In contrast, before infection, no tetramer+ cells in blood expressed either marker. However, during infection, a proportion of tetramer+ cells in blood upregulated CD103, peaking at day 10 then decreasing with resolution of infection. In BAL, the CD103 single-positive population also increased in frequency at day 10, transiently making up a greater proportion of tetramer+ cells, which then returned to a predominantly CD69+CD103+ phenotype during convalescence.

Analysis of CD45RA and CCR7 allowed further categorization of memory subsets and indicated that the majority of tetramer+ cells exhibited effector memory (Tem) or effector memory re-expressing CD45RA (Temra) phenotypes, with predominantly Tem cells in BAL (Fig. 6b). In blood, infection led to a significant increase in T-effector cells (expressing the CD45RA−/CCR7+ phenotype) at the expense of Temra cells, a change that was not seen in the airway. This occurred via proliferation starting between day 3 and day 7 post infection, paralleling the polyclonal CD8+ T-cell response and peaking at around day 10, when ∼85% (IQR 70–92%) of epitope-specific CD8+ T cells in blood showed activation and proliferation by CD38 and Ki-67 expression (Fig. 6c). This was also seen in tetramer+ BAL cells, although a proportion of these remained quiescent. However, by day 28, despite the continued accumulation of tetramer+ cells in the BAL, there was no evidence of on-going activation or proliferation.

**Figure 7:**
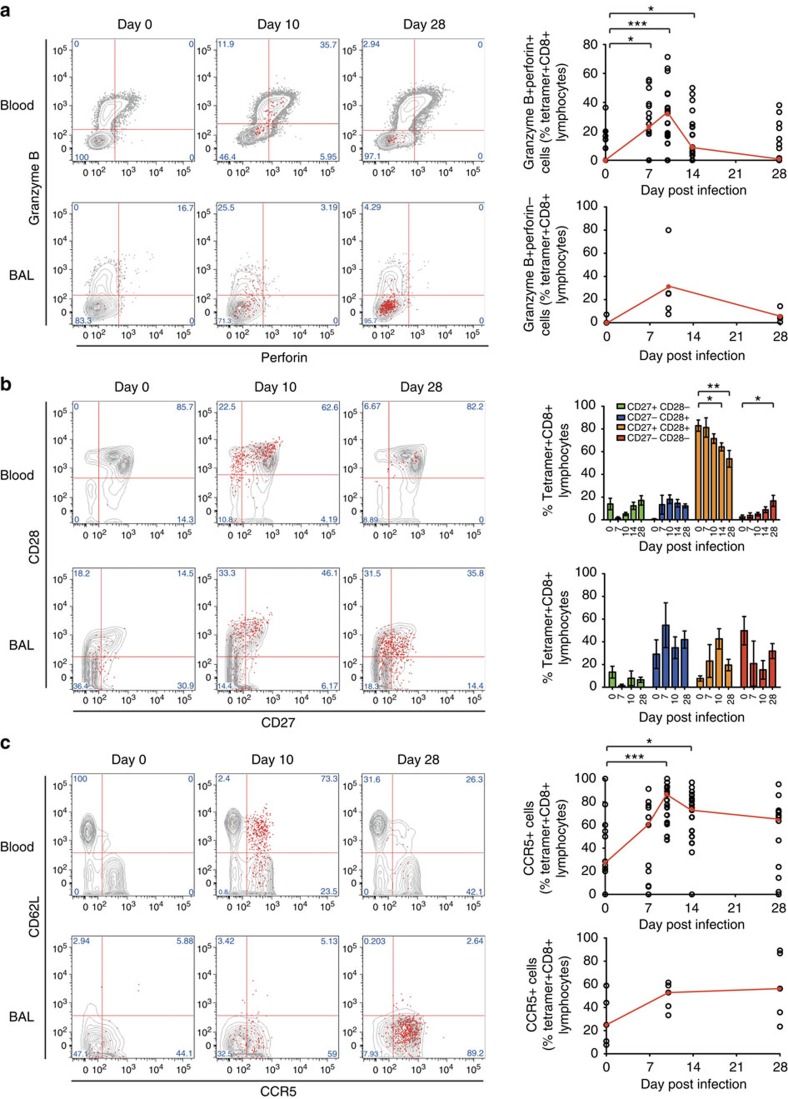
Reduced expression of cytotoxicity and co-stimulatory markers by RSV-specific CD8+ T cells in the airway. Tetramer+ CD8+ T cells in blood and BAL were co-stained for phenotypic markers. (**a**) Cytotoxicity molecules perforin and granzyme B are shown in blood (*n*=19) and BAL (*n*=5). Significant *P* values for two-tailed Wilcoxon matched pairs tests compared with baseline are shown in blood (day 7, *P*=0.0144; day 10, *P*=0.0009; and day 14, *P*=0.0413). (**b**) Co-stimulatory molecules CD27 and CD28 are shown in blood (*n*=19) and BAL (*n*=10). Mean±s.e.m. and significant *P* values for two-tailed Wilcoxon matched pairs tests compared with baseline are shown in blood for CD27+CD28+ cells (day 14, *P*=0.0454; day 28, *P*=0.0068) and CD27−CD28− cells (day 28, *P*=0.0144). (**c**) Homing receptors CCR5 and CD62L are shown in blood (*n*=19) and BAL (*n*=5). Significant *P* values for two-tailed Wilcoxon matched pairs tests compared with baseline are shown in blood (day 10, *P*=0.0009; day 14, *P*=0.0245). Throughout, representative plots from a single subject at day 0, 10 and 28 post infection are shown with tetramer+ cells as red dots and total CD8+ T cells in grey contours. Summarized data are shown for all RSV-infected subjects (who could be analysed using tetramers) with median frequencies shown in red. Abbreviated *P* values are shown: NS (not significant)=*P*>0.05, *P<0.05, **P<0.01.

In blood, these cells upregulated perforin and granzyme B, which also peaked around day 10 with around 33% (median, IQR 17–46%) expressing cytotoxicity molecules (Fig. 7a). However, few tetramer+ BAL cells expressed perforin. Furthermore, only a minority upregulated granzyme B, which again peaked around day 10. During infection, the co-stimulatory molecules CD27 and CD28 were both downregulated in a proportion of RSV-specific CD8+ T cells in the blood and in the majority of individuals had not returned to baseline frequencies by day 28 (Fig. 7b). In contrast, even before infection, a large proportion of RSV-specific CD8+ T cells had already downregulated CD28 and CD27+CD28+ double-positive cells were in the minority. Furthermore, there were no significant changes in their expression following infection. Finally, following activation, RSV-specific CD8+ T cells in blood upregulated CCR5 and downregulated CD62L, allowing homing to sites of inflammation and away from lymphoid tissues (Fig. 7c; Supplementary Fig. 3). In BAL, there was a trend towards tetramer+ Trm cells upregulating CCR5 and there was no CD62L expression at any time.

These data suggest that RSV infection induces a transient increase of virus-specific CD8+ T cells in the blood, with a phenotype characteristic of cytotoxic effector cells, some of which changes persist into the convalescent period. In contrast, Trm cells in the airway alter less markedly following infection, with a large proportion showing no proliferative response and overall reduced expression of cytotoxicity molecules.

### Limited functionality of RSV-specific CD8+ memory T cells

In studies of influenza, virus-specific CD8+ memory T-cell frequencies in the blood have been shown to correlate inversely with symptom severity during subsequent infection^15^,^16^. This has not been demonstrated with other viruses, and indeed we found no significant difference in baseline frequencies of RSV-specific CD8+ T cells in peripheral blood between those subsequently deemed infected and those who remained uninfected (Supplementary Fig. 4a). In addition, there was no statistical relationship between the magnitude of pre-existing HLA-restricted responses and the severity of disease, expressed throughout as cumulative viral load or symptom score to smooth out day-to-day variability in spite of the close correlation between peak and cumulative values (Supplementary Fig. 4b).

**Figure 8:**
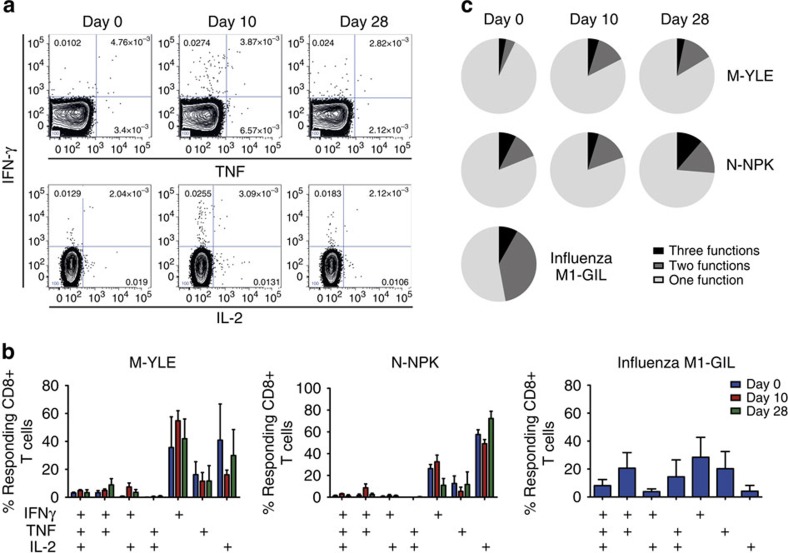
RSV-specific CD8+ T cells in blood show limited polyfunctionality. PBMCs from individuals inoculated with RSV were stimulated with peptide epitopes (YLE, *n*=3; NPK, *n*=4; and influenza M1-GIL, *n*=6) and subsequently intracellularly stained for IFN-γ, tumour necrosis factor (TNF) and interleukin-2 (IL-2) for analysis by flow cytometry. (**a**) Flow cytometric data gated on CD3+CD8+ lymphocytes from one representative donor are shown. Numbers represent percentage of CD8+ lymphocytes. (**b**) The mean (±s.e.m.) frequencies of cytokine-producing CD8+ T cells as determined by Boolean gating are shown as percentages of total responding cells. (**c**) The proportion of single, double and triple cytokine producers is shown.

Analysis of CD8+ T cells induced by highly protective systemic vaccines, such as yellow fever, have displayed polyfunctionality that may correlate with their efficacy^29^. To investigate whether the functionality of RSV-specific CD8+ T cells might be contributing to lack of protection, cytokine production was analysed by flow cytometry (Fig. 8a). Overall, the frequencies of cytokine-producing CD8+ T cells following short-term peptide stimulation were modest with average median frequencies of 0.035 (against YLE) and 0.056% (against NPK). These were similar to those seen against the immunodominant influenza epitope M1-GIL in A*02:01-expressing individuals from this cohort. However, most cytokine-expressing RSV-specific cells produced only a single cytokine (IFN-γ, tumour necrosis factor or interleukin-2) with an average of only 2.63% (±s.e.m. 0.432) producing three (Fig. 8b,c; Supplementary Fig. 5). By comparison, influenza-specific CD8+ T cells were more polyfunctional, with 39% producing two cytokines and a significantly higher proportion (15.1%±s.e.m. 2.30) producing three (*P*=0.0079 by Mann–Whitney test). Thus, RSV-specific memory CD8+ T cells in blood have reduced functionality compared with those against influenza, potentially contributing to reduced protective capacity.

### CD8+ Trm cells in airways correlate with reduced disease

**Figure 9:**
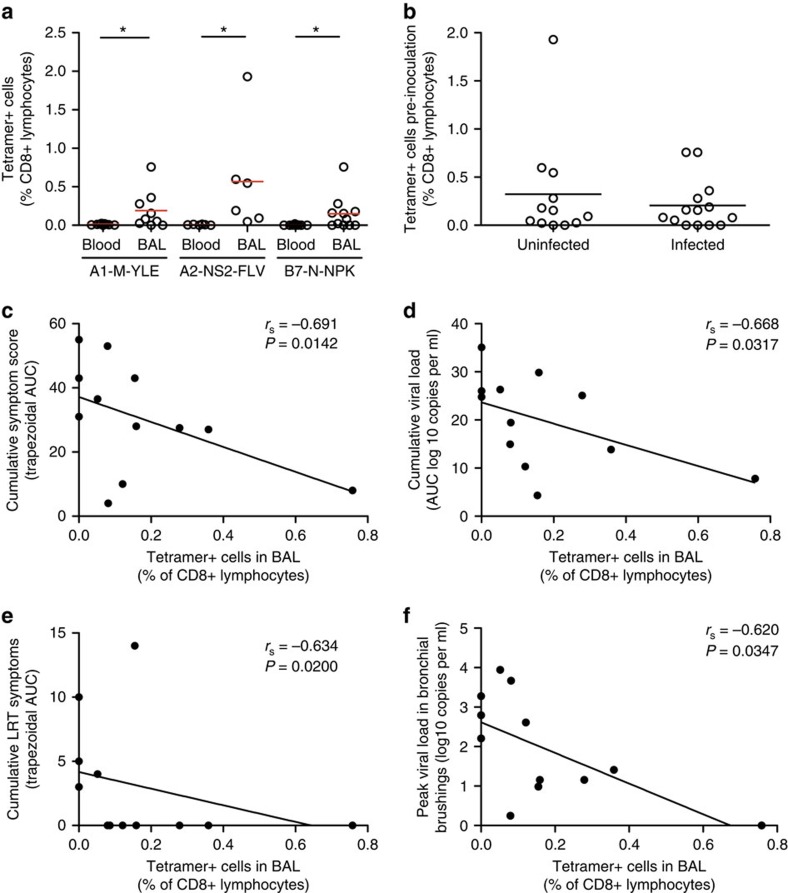
Pre-existing RSV-specific memory CD8+ T cells in the airway correlate with reduced disease severity. RSV-specific CD8+ T-cell responses to HLA-A*01:01-, A*02:01- and B*07:02-restricted epitopes were analysed by tetramer staining before inoculation. (**a**) Baseline frequencies of tetramer+ cells in infected HLA-A*01:01+ (*n*=9), A*02:01+ (*n*=6) and B*07:02+ (*n*=11) in matched blood and BAL samples were compared. The medians and *P* values for Wilcoxon matched pairs tests (A1-M-YLE, *P*=0.0234; A2-NS2-FLV, *P*=0.0313; B7-N-NPK, *P*=0.0195) are shown (*P<0.05). (**b**) Baseline frequencies of tetramer+ cells in BAL from infected (*n*=14) and uninfected (*n*=12) individuals were compared and tested for significant difference using the Mann–Whitney test (*P*=0.9256). Medians are shown. (**c**) Non-linear regression was used to assess the association between baseline RSV-specific CD8+ cell frequencies in BAL and disease severity in infected individuals as measured by (**c**) cumulative symptom score, (**d**) nasal viral load (trapezoidal area under the curve (AUC)), (**e**) cumulative lower respiratory tract symptoms and (**f**) peak viral load in bronchial brushings. Spearman's rank correlation coefficient (*r*_s_) and *P* values are shown.

These findings mirror our previous study that showed RSV-specific nasal IgA correlating strongly with protection from PCR-confirmed infection with greater predictive power than serum IgG in this regard^9^. However, where infection did occur, high nasal IgA titres did not reduce disease severity (Supplementary Fig. 6). We hypothesized that local cell-mediated immunity could provide the next layer of defence by acting to directly eliminate cell-associated virus and thus reduce symptoms. To test this, we analysed the frequencies of tetramer+ CD8+ T cells against immunodominant epitopes (representative of the RSV-specific CD8+ T-cell population before infection) and their relationship with infection risk and disease severity. At baseline, RSV-specific CD8+ Trm cells were already enriched in BAL with significantly higher frequencies compared with blood irrespective of specificity (Fig. 9a). However, in contrast to mucosal IgA, their frequency had no impact on the likelihood of PCR-confirmed RSV infection (Fig. 9b). Instead, the higher the frequency of RSV-specific CD8+ T cells in baseline BAL, the lower the cumulative symptom score (Spearman's *r*=−0.691, *P*=0.0142; Fig. 9c) and the lower the cumulative viral load (Spearman's *r*=−0.668, *P*=0.0317; Fig. 9d) in those who subsequently developed PCR+ infection. These correlations held true on correlation with lower respiratory tract symptoms alone (Fig. 9e, Spearman's *r*=0.634, *P*=0.02) but less so with upper tract or systemic symptoms (Supplementary Fig. 7). Similarly, higher frequencies of pre-existing CD8+ Trm cells correlated with lower bronchial viral load (Spearman's *r*=0.57, *P*=0.042; Fig. 9f), suggesting that these cells might have a direct role in viral clearance. In summary, while memory CD8+ T cells in blood do not correlate with protection against RSV, higher frequencies of CD8+ Trm cells in the airway are associated with improved viral control and reduced symptom severity, suggesting that these are likely to have a role in ameliorating disease if present in sufficiently high numbers.

## Discussion

Studies in animal models have increasingly highlighted the distinctiveness of local immunity and the importance of this compartmentalization to protective immune responses. Here we present the first interventional study to track virus-specific cell-mediated immunity in the human airway. Longitudinal analysis of the CD8+ T-cell response showed the extent to which these cells in the human airway diverge from those in the blood during RSV infection in almost every respect and provides strong evidence for the role that human Trm cells play in protection.

RSV caused surprisingly extensive inflammation in most infected individuals despite minimal or no lower respiratory tract symptoms. Compared with rhinovirus (where neither macroscopic inflammation nor cellular infiltrates are significantly seen on bronchoscopy in healthy adults^30^), RSV led to substantially greater lower respiratory tract involvement. Infection caused CD8+ T-cell infiltration of the respiratory mucosa with an unexpected accumulation of very large numbers of RSV-specific cells well into convalescence, distinct from blood in the same individuals. In both blood and airway, activation of CD8+ T cells occurred acutely, coinciding with falling viral load, suggesting their involvement in virus elimination. Shortly after peak viral shedding, proliferation ceased, implying that viral replication was required to drive this response.

In immunodeficient children, the absence of T cells is associated with prolonged and more severe infection, while RSV disease risk in older adults is believed to be related to fewer IFN-γ-producing T cells^14^,^31^. However, the low frequency, short-lived boosting and possibly limited functionality of RSV-specific memory CD8+ T cells in blood meant that poor correlation with protection was seen. In contrast, epitope-specific CD8+ Trm cells in the airways with their significantly higher frequencies and localization near the site of infection did indeed correlate with reduced disease severity. This strongly supports the hypothesis that virus-specific CD8+ Trm cells in the airway play a direct role in early clearance of respiratory viruses, while memory T cells in blood correlate indirectly (if at all). Previous studies in man, including those inferring the protective role of CD4+ and CD8+ T cells from peripheral blood in influenza^15^,^16^, make the assumption that there is a direct and proportional relationship between blood and mucosal immunity. Our findings show that this assumption is fundamentally incorrect and that these compartmentalized populations must be examined directly. Recent phase-I clinical trials have shown that virus-vectored RSV vaccine candidates can induce T cells in blood^32^. Our data suggest that their capacity to induce Trm cells following intranasal administration should be explored further. These findings might also explain why CD8+ T-cell-inducing influenza vaccines that induce primarily systemic responses demonstrate suboptimal efficacy^5^,^33^.

In mice, expression of the receptors CD69 and CD103 allows retention of CD8+ Trm in tissues^34^. Although they may not represent the entire Trm population^35^, CD69+CD103+ CD8+ Trm cells have been shown to localize to the murine airway following respiratory priming with influenza and mediate cross-protection against heterologous strains^21^. Much less is known in humans, where previous studies have been based solely on uninfected lung tissue removed during cancer surgery or non-living organ donations and limited to cross-sectional analysis of resting T cells^22^,^23^. These estimates of frequency showed that Trm cells are abundant in the lung where, at rest, influenza-specific CD8+ Trm cells are more frequent than those for cytomegalovirus. Our longitudinal analyses advance these findings by showing *in vivo* how Trm cells in the human airway arise following viral infection and how they relate to disease severity. During acute infection, RSV-specific CD8+ Trm cells were transiently joined by a CD103+CD69− population that could represent a population that migrate from blood to airway, although the ontogeny of Trm cells has yet to be fully elucidated^36^,^37^. Interestingly, this may be the reverse of CD8+ Trm cells generated against herpes simplex virus 1 (HSV-1) in murine skin, where sequential induction of first CD69 and then CD103 has been observed, suggesting the impact of tissue type, pathogen and/or interspecies differences^38^.

RSV-specific Trm cells in the airway showed phenotypic changes suggestive of advanced differentiation, with downregulation of co-stimulatory markers, but also reduced expression of cytotoxicity molecules. We therefore propose that the very high Trm cell frequencies seen during early convalescence can confer absolute protection against early symptomatic reinfection but may be functionally regulated to prevent excessive damage to the delicate lung architecture. Inflammatory changes seen in the airway during convalescence may represent the effect of large numbers of CD8+ Trm cells interacting with residual RSV antigen, but these caused no symptoms in healthy adults. In contrast, in murine systems where large numbers of RSV-specific CD8+ T cells are present at the same time as high viral titres, life-threatening immunopathology can occur^39^. In our study, it was not deemed acceptable to subject volunteers to a fourth bronchoscopy to determine the longevity of Trm cells, while other phenotypic and functional assessments were likely to be qualitatively similar to the preinfection time point. Nevertheless, it can be inferred that in these volunteers, CD8+ Trm frequencies had waned significantly, since the end of their last natural RSV infection (though less precipitously than in blood). This waning in the lung contrasts with long-lived Trm cells in other tissues and may represent another mechanism by which immunopathology is reduced^40^. Outstanding questions including how Trm cells differentiate and the conditions required for persistence remain major hurdles for generating protective cell-mediated immunity in the mucosa. Both residual antigen and cytokines such as TGF-β have been implicated in Trm cell maintenance. Blockade of antigen recognition in the lungs of influenza-infected mice leads to loss of Trm cells, while TGF-β is expressed in respiratory mucosa and *in vitro* can induce upregulation of CD103 (ref. 41). This is supported by our findings of persistent RSV antigen in the airway associated with virus-specific CD8+ Trm cells even 28 days after inoculation. While there was no apparent disease associated with high frequencies of RSV-specific CD8+ Trm cells in our cohort, damaging immunopathology caused by boosting them in other age groups and settings remains a risk.

These data show that immune responses in blood can fundamentally misrepresent those at the site of infection. Our *ex vivo* analyses of the respiratory compartment show that adaptive immunity against respiratory viruses comprises multiple specialised and non-redundant protective mechanisms distinct in time and spatial organization. In RSV, locally produced mucosal IgA constitutes an initial barrier to virus entry but does not significantly modulate disease severity once the barrier is breached^9^. When that occurs, CD8+ Trm cells provide early recognition and virus elimination, thus reducing symptom severity and viral load. Only after these does systemic immunity act to prevent widespread disease. We therefore propose that Trm cells represent one of several immune mechanisms that should be harnessed together for optimal vaccine-mediated protection.

## Methods

### Ethics statement

The study was approved by the UK National Ethics Service London—Fulham (study numbers 10/H0711/94 and 11/LO/1826). Written informed consent was obtained from all volunteers.

### Study design

Healthy, non-smoking adults aged 18–55 years were recruited between 2012 and 2013. All subjects were genotyped for HLA class-I loci using sequence-based typing. A total of 49 subjects were inoculated with 10^4^ plaque-forming units of RSV Memphis 37 (Meridian Lifesciences, Memphis, USA). Following the challenge, participants were quarantined for 10 days. Cold symptoms were assessed using symptoms diaries completed daily by participants. Reported symptoms with a score of 0 (absent), 2 (moderate) or 3 (severe) included sneezing, nasal discharge, nasal obstruction or sore throat (upper respiratory tract), headache, malaise, fever (systemic) and cough, wheeze, shortness of breath (lower respiratory tract symptoms). Cold was confirmed if two out of the following three conditions were fulfilled: nasal discharge lasting ≥3 days, subjective feeling of cold reported or cumulative 14-day symptom score ≥14. Blood samples were obtained at baseline and time points post inoculation as indicated in the text. Nasal lavage samples were collected daily and used for viral load quantification by qPCR as previously described^9^,^42^. Briefly, total RNA was isolated using the QIAamp Viral RNA kit (Qiagen) according to the manufacturer's instructions. Reverse transcription of 13 μl of total isolated RNA was achieved using the High Capacity RNA-to-cDNA kit (Applied Biosystems) according to the manufacturer's instructions. qPCR with reverse trancription reactions for RSV N gene were achieved using primers shown in Supplementary Table 7 and the TaqMan Universal Master Mix II (Applied Biosystems) and 7500 Fast Real-Time PCR System (Applied Biosystems). Absolute quantification was calculated using a plasmid DNA standard curve. As shown previously, infection status as determined by qPCR positivity accorded 100% with multiplex qualitative PCR and plaque assay. Bronchoscopists were blinded to the infection status of the volunteers. Bronchoscopies were undertaken at 14 days before inoculation and 7 or 10 days and 28 days post infection. Macroscopic appearance of the airways was reported as ‘normal', ‘erythematous' or ‘erythematous with contact bleeding'. Bronchial brushings and biopsies were obtained at each time point. Bronchial epithelial cells were washed with RPMI/10% fetal calf serum (FCS; R10) and stored in TRIzol reagent (Invitrogen, Grand Island, USA) for viral load quantification. BAL was obtained by instillation of up to 120 ml of normal saline, filtered and centrifuged before flow cytometric analysis.

### Immunohistochemistry

Endobronchial biopsies were fixed immediately in 4% paraformaldehyde and paraffin embedded. RSV was stained using polyvalent mouse anti-RSV antibody (NCL-RSV3, Leica Biosystems, UK) at 1:50 dilution. CD8+ T cells were identified by staining with mouse anti-CD8 (M0707, Dako) at 1:100 dilution using the EnVision peroxidase staining method (Dako, Denmark) as previously described^30^. Briefly, 5-μm sections were stained according to the manufacturer's instructions and an irrelevant mouse IgG1 kappa antibody (MOPC21) was used as negative control for staining specificity of mouse monoclonal antibodies. RSV-infected A549 cells were used as positive staining controls. Quantification was achieved as previously described with operators blinded to sample timing and infection status^30^. Briefly, slides were coded to avoid observer bias and areas of epithelium and subepithelium assessed using a Leitz Dialux 20 light microscope, Apple Macintosh computer and Image 1.5 software. Total epithelial and subepithelial areas of two to three bronchial biopsies were counted for each bronchoscopy. Cell counts were expressed as the number of cut cell profiles with visible nucleus per mm^2^ of subepithelium and per 0.1 mm^2^ of epithlium. The coefficient of variation for repeat counts of positive cells by a single observer ranged from 5 to 6%.

### PBMC isolation

PBMC isolation was performed by density centrifugation using Histopaque 1077 (Sigma Aldrich, USA) according to the manufacturer's protocol. Cells were either used immediately or cryopreserved in FCS (Gibco Life Technologies, USA) with 10% dimethyl sulfoxide (DMSO) in liquid nitrogen for future assays.

### *Ex vivo* interferon-γ ELISpot assay

T-cell epitope mapping was performed by INF-γ ELISpot using 2 × 10^5^ PBMCs in triplicate stimulated with peptide pools (10 μg ml^−1^ of each peptide) for initial screening followed by deconvolution with single peptides (10 μg ml^−1^). Plates were incubated for ≥17 h at 37 °C and subsequently developed (Mabtech, Sweden). SFU were counted using AID ELISpot software (Autoimmun Diagnostika GmbH, Germany) and analysed with positive wells containing ≥20 spots per 10^6^ cells and a *P* value of ≤0.05 using a Student's *t*-test in at least two experiments.

### Flow cytometry

HLA class-I (A*01:01 and B*07:02) tetramers were a gift from Rafi Ahmed (Emory University, Atlanta, USA). Custom-made dextramers (A*02:01) were purchased from Immudex (Copenhagen, Denmark). Whole blood or BAL cell samples were stained by adding 2 μl of tetramer and incubating for 10 min at room temperature before staining with surface antibodies for 20 min. Dextramer staining was performed in the same way on freshly isolated PBMCs or BAL cells. Surface staining of whole blood was followed by erythrocytes lysis with BD Lysis buffer. Cells were then fixed with 1% paraformaldehyde solution or prepared for intracellular staining by fixing and permeabilization with BD Fixation/Permeabilization kit. The following surface antibodies (including clone and catalogue number) were used in the study: CD3 PE-CF594 (UCHT1, BD#562280), CD4 APC-H7 (SK3, BD#641398), CD38 PE Cy7 (HB7, BD#335825), Ki-67 FITC (B56, BD#556026), Perforin FITC (δG9, BD#556577), Granzyme B V450 (GB11, BD#561151), CD45RA FITC (HI100, BD#555488), CCR7 PE (150503, BD#560765), CD27 V450 (M-T271, BD#560448), CD28 PE Cy7 (CD28.2, BD#560684), CCR5 V450 (2D7/CCR5, BD#562121), CD62L PE (DREG-56, BD#555544; all BD Biosciences); CD8 PerCP Cy 5.5 (RPA-T8, #45-0088-42), CD69 FITC (FN50, 11-0699-42) and CD103 PE Cy7 (B-Ly7, #25-1038-42; all eBioscience). Dilutions at which antibodies were used are shown in Supplementary Table 8. FlowJo software (FlowJo LLC, USA) was used for data analysis.

### *In vitro* peptide stimulation and intracellular cytokine staining

Intracellular cytokine staining was used to confirm epitopes identified by ELISpot. Thawed PBMCs were rested overnight and stimulated the next day for 6 h in culture medium (RPMI 1640, 10% FCS, 2 mM glutamine, 100 IU ml^−1^ penicillin/streptomycin) using selected peptides (10 μg ml^−1^) plus anti-CD28 and anti-CD49. After 2 h, Brefeldin A (1 μl/ml) was added and after 6 h cells were washed and stained with live/dead marker (Invitrogen, USA); IFN-γ APC (B27, #554702), TNF PE Cy7 (MAb11, #557647), interleukin-2 FITC (5344.111, #340448; all BD Biosciences); and relevant surface markers. Dilutions at which antibodies were used are shown in Supplementary Table 8. The frequencies of expression profiles for single, double and triple cytokine producers were calculated using the Boolean combination gating option in FlowJo software (FlowJo LLC, USA).

### RSV Memphis 37 sequence translation and *in silico* epitope prediction

The genomic sequence of RSV Memphis 37 was translated into proteins from all six possible open-reading frames and the resulting amino-acid sequences compared against 11 RSV reference protein sequences obtained from RefSeq using blastx. Hits were filtered by removing those with an e-value lower than 1 × 10^−6^. These were then verified by manual inspection. The capacity of all 9- and 10-mer peptides derived from RSV M37 to bind HLA-A*01:01, A*02:01 and B*07:02 was predicted with the Stabilized Matrix Method using the command-line version of the Immune Epitope Database consensus prediction tool^26^. Peptides were selected if they scored in the top 1% of predictions for each length or if they were the top two peptides for the protein (to ensure even small proteins were represented for each allele). Peptides were synthesized by A&A Labs LLC (San Diego, CA, USA) as crude material, and resuspended at 20 mg ml^−1^ in 100% DMSO (v/v).

### MHC peptide-binding assays

Quantitative measurements of peptide binding to HLA class-I molecules were made by inhibition of binding of radiolabelled standard peptides. MHC molecules were purified by affinity chromatography from the Epstein–Barr virus-transformed homozygous cell line JY, and assays performed, as described previously^43^. Briefly, peptides were tested at six different concentrations covering a 100,000-fold dose range in three or more independent assays. Lyophilized test peptides were solubilized in water, PBS (pH 7.2) or 100% DMSO and serially diluted in 0.05% (v/v) NP-40/PBS. Each peptide dose (5 μl) was loaded into the reaction vessel (Costar 96-well polypropylene round-bottom plate). For positive (no peptide) and negative (no MHC) controls, wells were loaded with NP-40/PBS alone. The MHC/labelled peptide reaction mix was prepared with PBS, purified MHC of the desired allele, protease inhibitor cocktail, β_2_ microglobulin and radiolabelled peptide. Reaction mix (10 μl) was added to all but the negative control well and the plate sealed and incubated for 2 days in the dark at room temperature. The concentration of peptide yielding 50% inhibition of the binding of the radiolabelled probe peptide (half-maximal inhibitory concentration (IC_50_)) was calculated. Under the conditions used, where [radiolabelled probe]<[MHC] and IC_50_≥[MHC], the measured IC_50_ values are reasonable approximations of the true *K*_d_ values^44^,^45^.

### Statistical analysis

On the basis of published data^25^, we calculated that a minimum sample size of 16 in each arm (infected versus uninfected) would be sufficient to find a difference of 1.3% of epitope-specific CD8+ T cells with 80% power using a two-sided unpaired *t*-test with 5% significance level (where the variability in each group was 1.4%). Samples were only excluded where insufficient cells or material remained. Statistical analysis was performed using Graphpad Prism and R software.

## Additional information

**How to cite this article:** Jozwik, A. *et al.* RSV-specific airway resident memory CD8+ T cells and differential disease severity after experimental human infection. *Nat. Commun.* 6:10224 doi: 10.1038/ncomms10224 (2015).

## Supplementary Material

Supplementary Information Supplementary Figures 1-7 and Supplementary Tables 1-8
