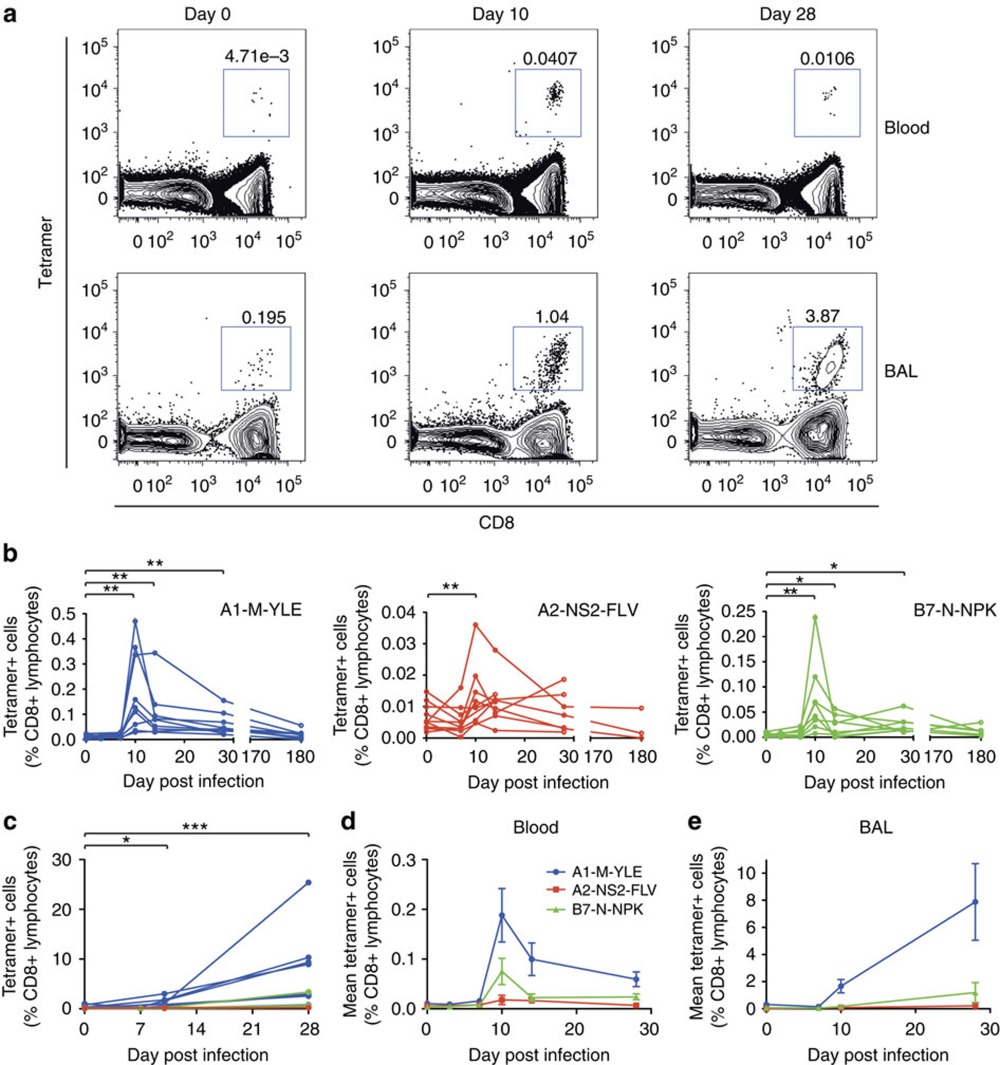# Erratum: RSV-specific airway resident memory CD8+ T cells and differential disease severity after experimental human infection

**DOI:** 10.1038/ncomms11011

**Published:** 2016-03-09

**Authors:** Agnieszka Jozwik, Maximillian S. Habibi, Allan Paras, Jie Zhu, Aleks Guvenel, Jaideep Dhariwal, Mark Almond, Ernie H. C. Wong, Annemarie Sykes, Matthew Maybeno, Jerico Del Rosario, Maria-Belen Trujillo-Torralbo, Patrick Mallia, John Sidney, Bjoern Peters, Onn Min Kon, Alessandro Sette, Sebastian L. Johnston, Peter J. Openshaw, Christopher Chiu

Nature Communications 6: Article number: 10224 10.1038/ncomms10224 (2015); Published: 12212015; Updated: 03092016

In Fig. 5 of this article, the three dot plots in the first row of panel **a** contain black numerical labels that are incorrect, and light blue labels that are correct. A revised version of Fig. 5, with correct labels in black throughout, appears below.

**Figure 5**