# Supplementary material for: RSV-specific airway resident memory CD8+ T cells and differential disease severity after experimental human infection
Source: Nat Commun. 2015 Dec 21;6:10224. doi: 10.1038/ncomms10224 (PMC4703893; doi:10.1038/ncomms10224)
Supplement: Supplementary Information — Supplementary Figures 1-7 and Supplementary Tables 1-8 [file ncomms10224-s1.pdf]

Supplementary Figure 1: Viral load correlates with symptoms severity following RSV M37 infection. Twenty-six subjects were infected with RSV M37. Viral load was measure by qPCR and symptoms assessed using self-reported symptom diaries. Cumulative scores were calculated using trapezoidal area under the curve (AUC).

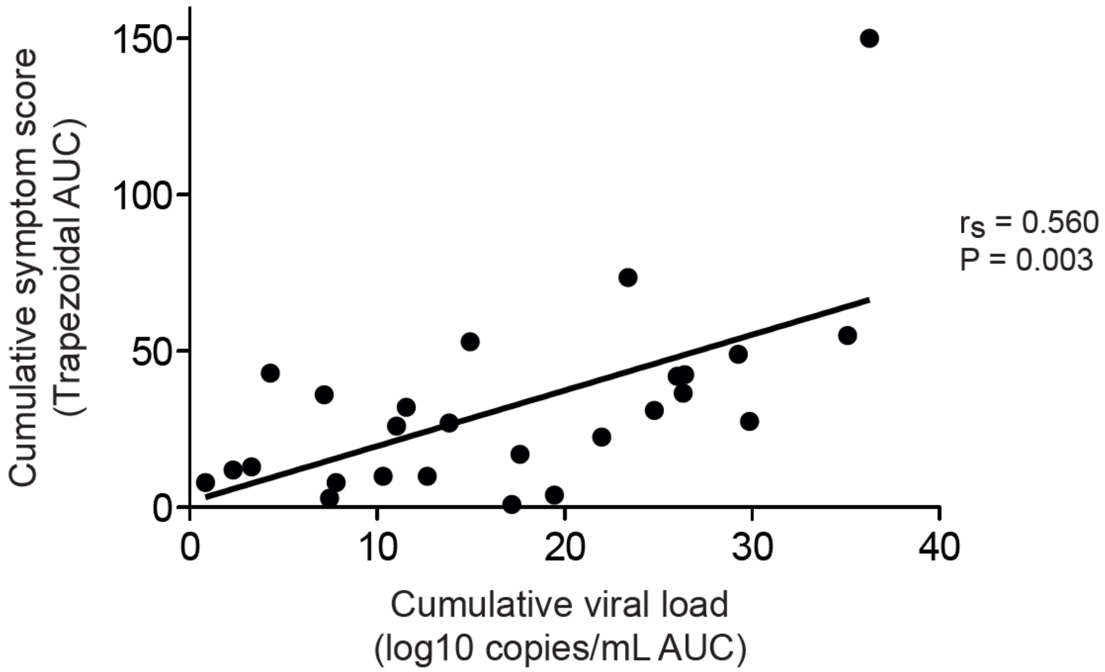

Supplementary Figure 2: Fold change in RSV-specific CD8+ T cells following infection. RSV-specific CD8+ T cells were enumerated by IFN-γ ELISpot at baseline and 10 days post-infection. The fold-changes in total responses are shown for each HLA allele.

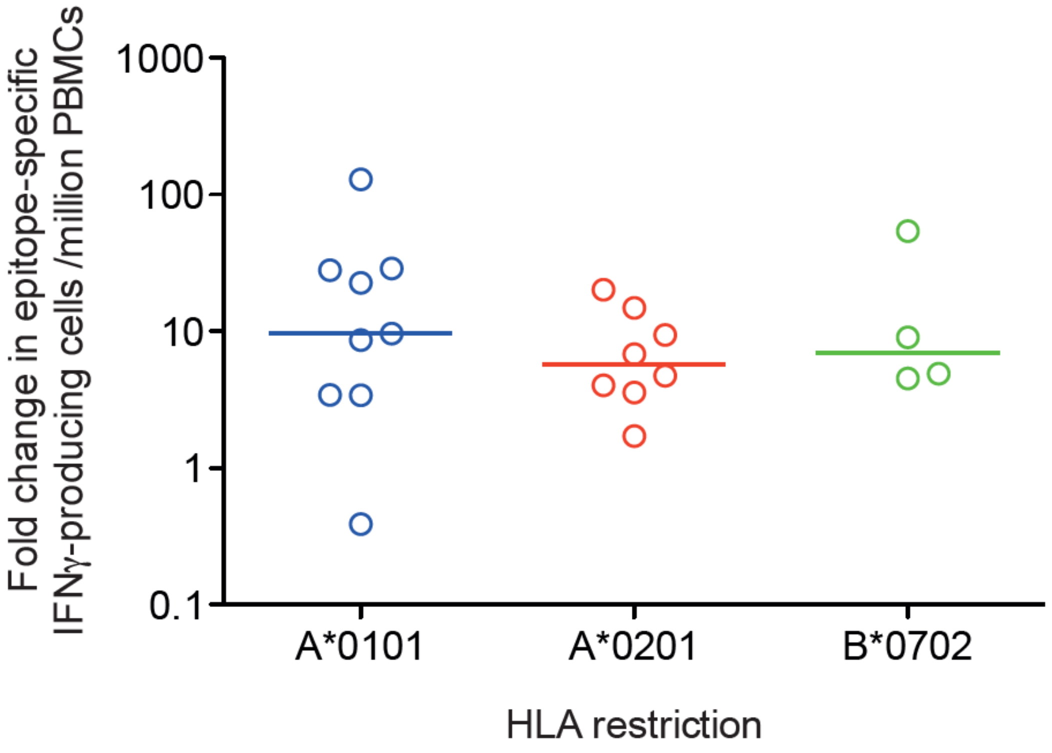

Supplementary Figure 3: RSV-specific CD8+ T cells downregulate CD62L on infection. Epitope-specific CD8+ T cells were co-stained with tetramer, anti-CD3, CD8 and CD62L. P-value for Wilcoxon matched pairs test is shown (*** = P < 0.001).

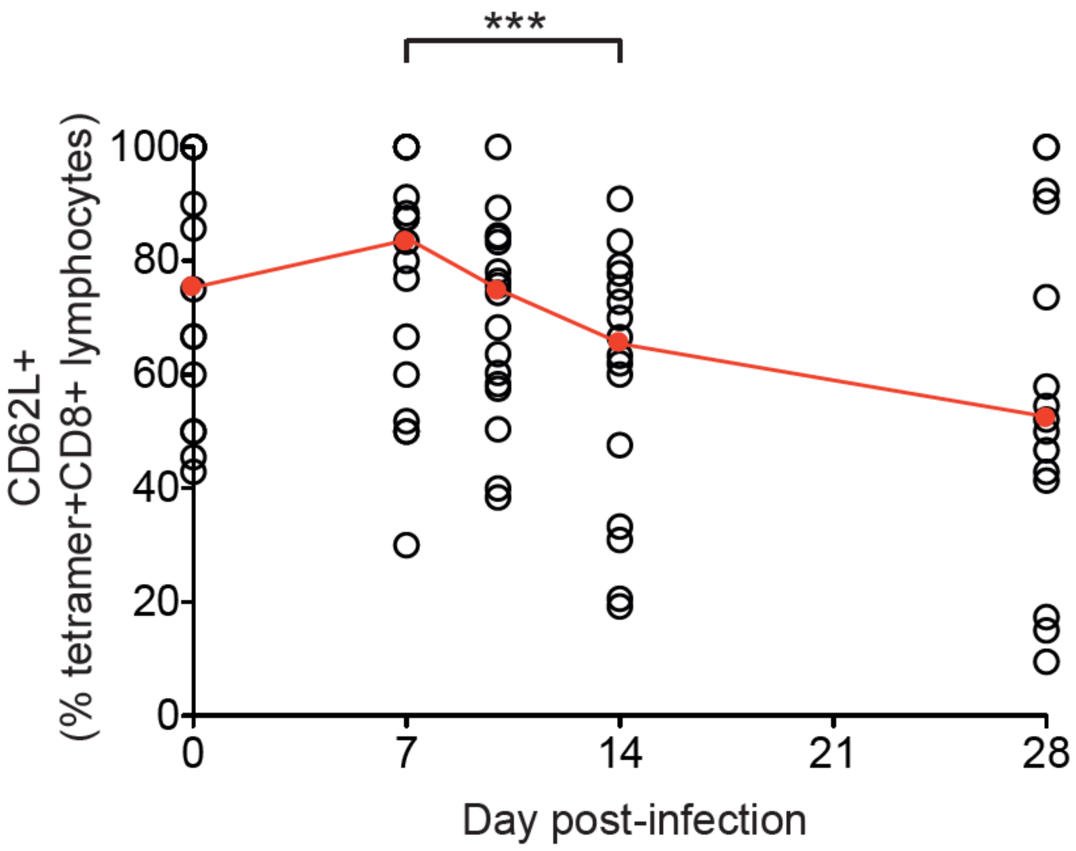

Supplementary Figure 4: Pre-existing RSV-specific memory CD8+ T cells in peripheral blood do not correlate with protection against repeated infection or severity of disease. RSV-specific CD8+ T cell responses to HLA-A*01:01, A*02:01 and B*07:02-restricted epitopes were analysed by IFN-γ ELISpot prior to inoculation. (a) The medians and P-values for Mann-Whitney tests (ns = P > 0.05) are shown. (b) Non-linear regression was used to assess the association between baseline RSV-specific CD8+ cell frequencies and disease severity as measured by cumulative viral shedding (trapezoidal area under the curve). Spearman's rank correlation coefficient (rs) and P values are shown. (c) The correlation between peak and cumulative viral load and symptoms is shown.

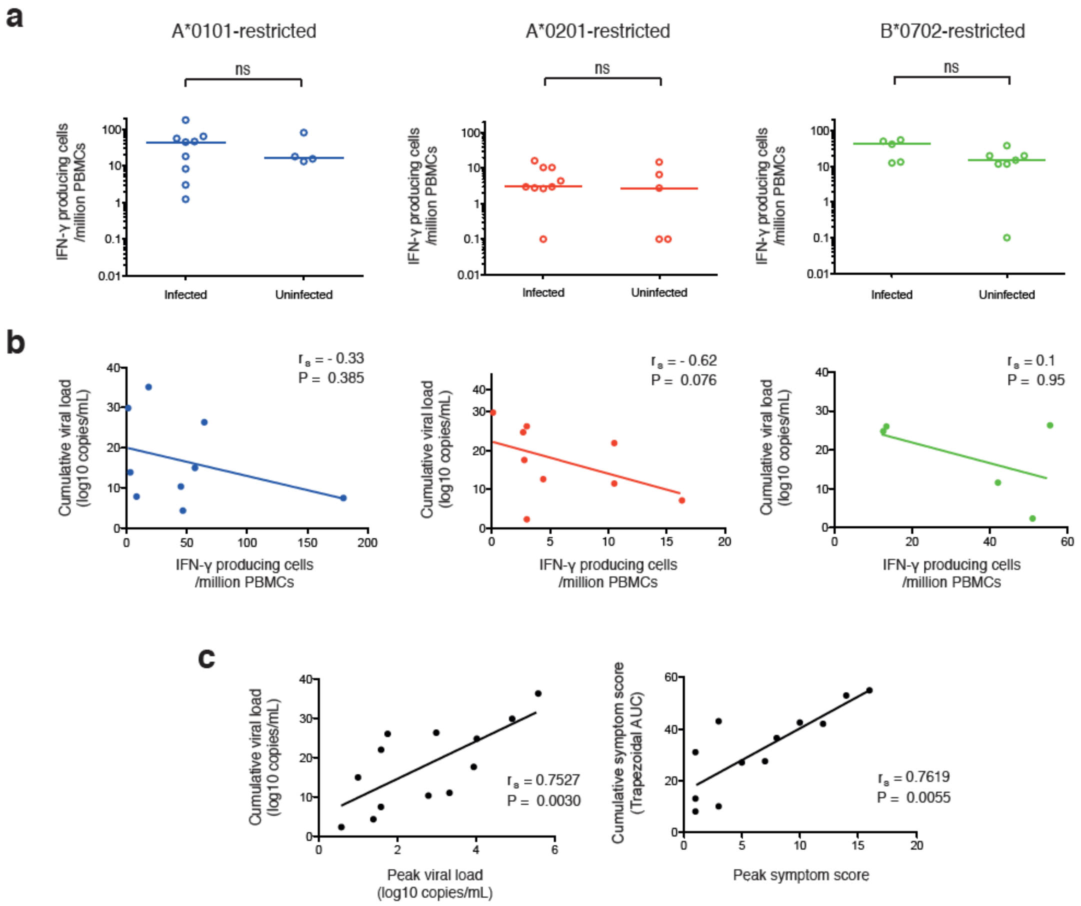

Supplementary Figure 5: RSV-specific CD8+ T cells are significantly less polyfunctional than influenza-specific CD8+ T cells. Resting PBMCs from individuals subsequently inoculated with RSV were stimulated with peptide epitopes (YLE, NPK and influenza M1-GIL) and subsequently intracellularly stained for IFN-γ, TNF and IL-2 for analysis by flow cytometry. The frequencies of CD8+ T cells co-expressing all three cytokines as determined by Boolean gating are shown as percentages of total responding cells.

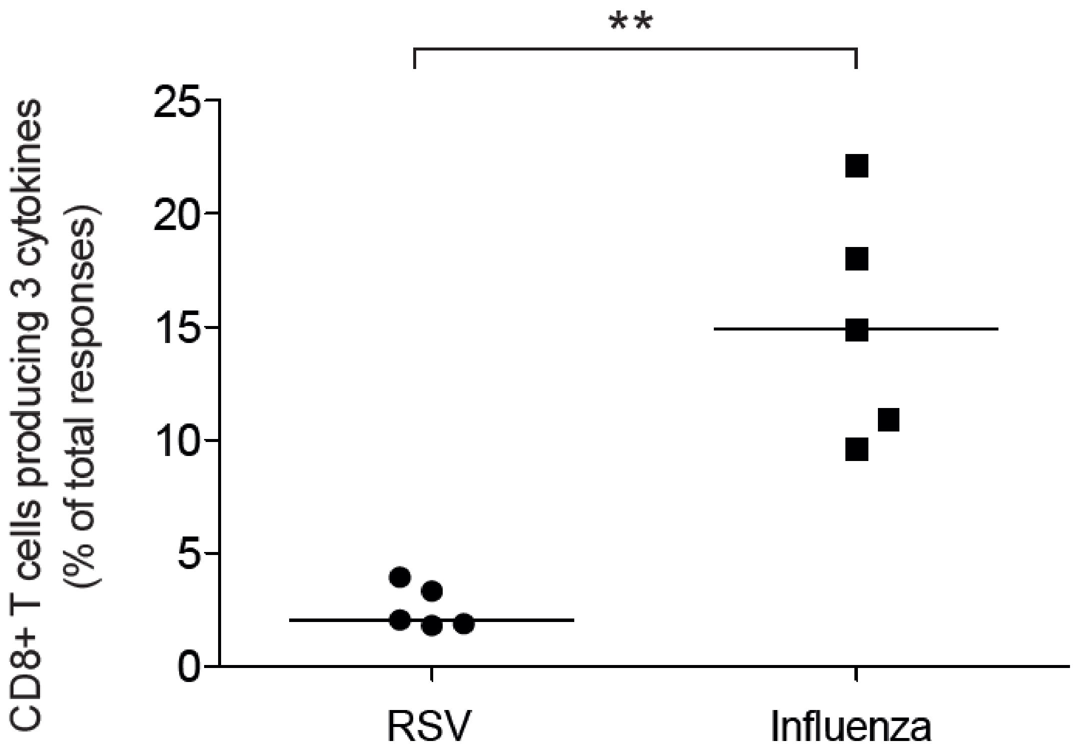

Supplementary Figure 6: RSV-specific nasal IgA correlates with protection from infection but not disease severity. RSV-specific IgA was analyzed by ELISA in nasal lavage fluid prior to experimental inoculation of volunteers. (a) The medians and P-value for the Mann-Whitney test (P=0.0316) are shown for individuals with and without PCR-confirmed infection. (b) Non-linear regression was used to assess the association between baseline RSV-specific nasal IgA titers and disease severity as measured by cumulative symptoms and viral shedding (trapezoidal area under the curve). Spearman's rank correlation coefficient (rs) and P values are shown.

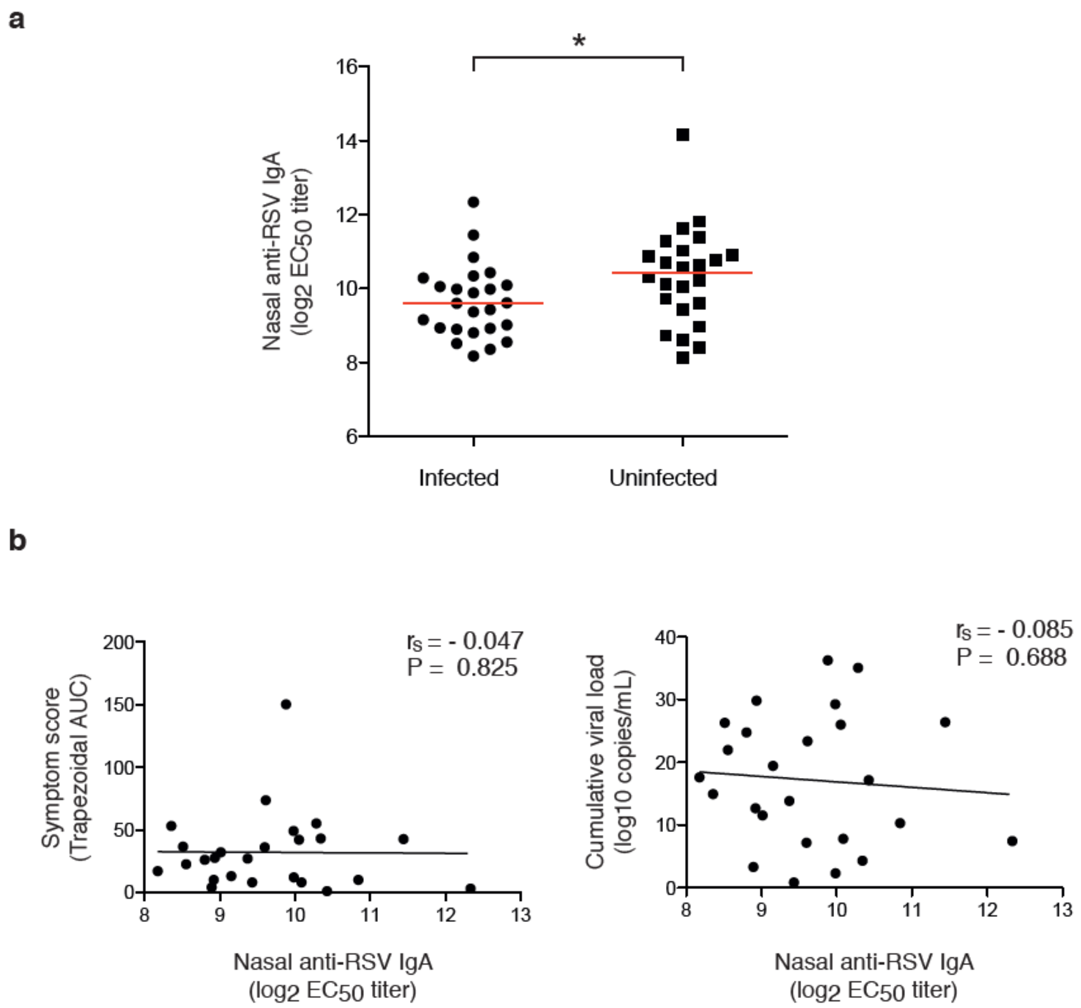

Supplementary Figure 7: Pre-existing RSV-specific CD8+ T cell frequency in the lower airway correlates loosely with upper respiratory tract and systemic symptoms. RSV-specific CD8+ T cell responses to HLA-A*01:01, A*02:01 and B*07:02-restricted epitopes were analysed by tetramer staining prior to inoculation. Non-linear regression was used to assess the association between baseline RSV-specific CD8+ cell frequencies in BAL and disease severity in infected individuals as measured by cumulative (a) lower respiratory tract and (b) systemic symptoms. Spearman's rank correlation coefficient (rs) and P values are shown.

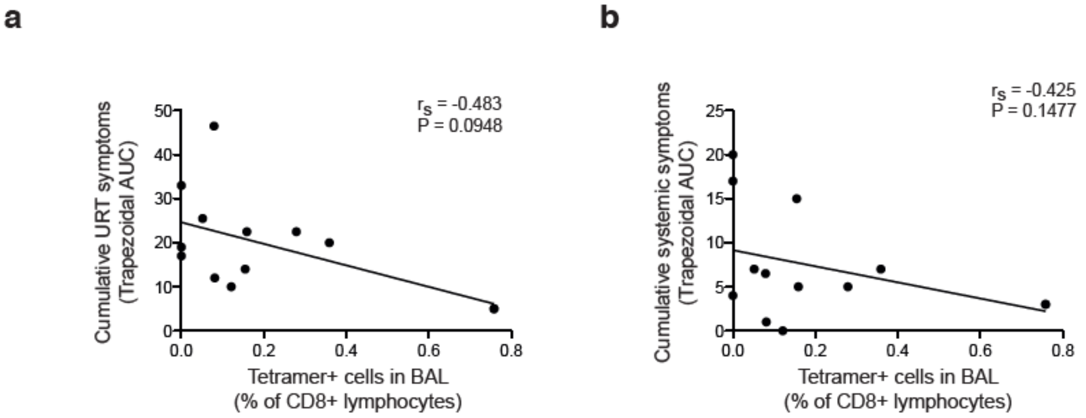

Supplementary Table 1: Demographic data summary of subjects inoculated with RSV

RSV PCR	No	Age (Median & range)	% Male	A*0101	A*0201	B*0702	A*0101 + A*0201	A*0101 + B*0702	A*0201 + B*0702	All 3 HLA alleles	Ethnicity %
PCR+	26	20.5 (18 – 50)	58	4	5	2	1	1	4	2	White 84.6<br>Black 3.8<br>Chinese 7.7<br>Mixed 3.8
PCR-	23	23 (18 – 39)	65	1	6	3	0	3	2	0	White 73.9<br>Black 17.3<br>Mixed 8.7

Supplementary Table 2: Summary of demographic and HLA typing data of volunteers inoculated with RSV and bronchoscopied

RSV PCR	No.	Age (Median & range)	% Female	Ethnicity	HLA-A*0101	HLA-A*0201	HLA-B*0702
Nasal PCR+	12	20 (18-50)	33.3	White 92%<br>Black 0%<br>Chinese 8.3%	8	3	6
Bronchial PCR+	4	23 (20-27)	25	White 100%	0	3	3
PCR-	8	23 (18-39)	16.7	White 75%<br>Black 25%<br>Chinese 0%	2	5	6

Supplementary Table 3: Macroscopic appearance of airways at bronchoscopy

	% Baseline		% Day 7		% Day 10		% Day 28	
	+	++	+	++	+	++	+	++
Nasal PCR+	0 (0/12)	0 (0/12)	42.9 (3/7)	0 (0/7)	60 (3/5)	40 (2/5)	10 (1/10)	40 (4/10)
Bronchial PCR+	0 (0/4)	0 (0/4)	100 (1/1)	0 (0/1)	0 (0/3)	33.3 (1/3)	0 (0/3)	0 (0/3)
PCR-	12.5 (1/8)	0 (0/8)	0 (0/4)	0 (0/4)	50 (2/4)	25 (1/4)	0 (0/8)	0 (0/8)

+ = erythema only, ++ = erythema plus contact bleeding/friability

Supplementary Table 4: RSV antigen detection by immunohistochemistry

	RSV Antigen+ IHC			
	% Baseline	% Day 7	% Day 10	% Day 28
Nasal PCR+	0 (0/11)	33.3 (2/6)	20 (1/5)	33.3 (3/10)
Bronchial PCR+	0 (0/4)	0 (0/1)	0 (0/3)	25 (1/4)
PCR-	0 (0/8)	0 (0/4)	0 (0/4)	9.1 (1/11)

Numbers indicate individuals with a staining score of 3-4 (on a 0-4 scale).

Supplementary Table 5: MHC class I restricted epitopes defined by epitope mapping of RSV Memphis 37

HLA restriction	Peptide ID	Position	Sequence	Conserved with RSV A2	Predicted affinity IC50 (nM)	Measured affinity IC50 (nM)
A*0101	NS1_2	115	KLSDSTMTNY		888	3835
A*0101	NS1_3	116	LSDSTMTNY		8	37
A*0101	M_2	205	VTDNKGAFKY	+	24	507
A*0101	M_7	228	YLEKESIYY	+	24	120
A*0201	NS2_3	60	FLVNYEMKL	+	9	349
B*0702	N_2	254	QVMLRWGVL	+	142	93
B*0702	N_8	305	NPKASLLSL	+	25	148
B*0702	G_2	239	KPNIRTTLL		13	73
B*0702	L_3	1352	IPAYRTTNY	+	451	1745

Supplementary Table 6: Antigen-specific CD8+ T cell responses by Interferon-γ ELISpot following experimental human RSV infection

	**A*01:01**			**A*02:01**			**B*07:02**		
	**Interferon-γ producing cells /million PBMCs**								
**Subject**	**Day 0**	**Day 10**	**Day 28**	**Day 0**	**Day 10**	**Day 28**	**Day 0**	**Day 10**	**Day 28**
1	65	623	141	3	28	7	56	272	63
2	1	159	67	0	6.67	10.67	5	0	37
3	8	240	69	N/A	N/A	N/A	7	0	38
4	180	70	60	N/A	N/A	N/A	N/A	N/A	N/A
5	45	389	38	N/A	N/A	N/A	N/A	N/A	N/A
6	47	159	52	N/A	N/A	N/A	N/A	N/A	N/A
7	57	194	7	N/A	N/A	N/A	N/A	N/A	N/A
8	18	514	83	N/A	N/A	N/A	N/A	N/A	N/A
9	3	68	17	N/A	N/A	N/A	N/A	N/A	N/A
10	N/A	N/A	N/A	4	18	16	N/A	N/A	N/A
11	N/A	N/A	N/A	11	50	7	N/A	N/A	N/A
12	N/A	N/A	N/A	3	42	20	N/A	N/A	N/A
13	N/A	N/A	N/A	16	58	20	N/A	N/A	N/A
14	N/A	N/A	N/A	3	20	28	51	397	142
15	N/A	N/A	N/A	3	54	5	13	678	117
16	N/A	N/A	N/A	10	18	5	42	190	52
17	N/A	N/A	N/A	0	0	0	13	121	13

Supplementary Table 7: Primers used for RSV quantification by PCR

Primer	Sequence
RSV A N-gene forward	5'-CATCCAGCAAATACACCATCCA-3'
RSV A N-gene reverse	5'-TTCTGCACATCATAATTAGGAGTATCAA-3'
RSV PAN A N-gene probe	5'-FAM-CGGAGCACAGGAGAT-TAMRA-3'

Supplementary Table 8: Antibodies used for flow cytometry

Antibody	Company	Clone	Catalogue number	Dilution
CD3 PE CF594	BD	UCHT1	562280	1:300
CD4 APC H7	BD	SK3	641398	1:150
CD38 PE Cy7	BD	HB7	335825	1:150
Ki67 FITC	BD	B56	556026	1:150
Perforin FITC	BD	δG9	556577	1:30
Granzyme B V450	BD	GB11	561151	1:60
CD45RA FITC	BD	HI100	555488	1:60
CCR7 PE	BD	150503	560765	1:30
CD27 V450	BD	M-T271	560448	1:150
CD28 PE Cy7	BD	CD28.2	560684	1 :150
CCR5 V450	BD	2D7/CCR5	562121	1:60
CD62L PE	BD	DREG-56	555544	1:60
CD69 FITC	eBioscience	FN50	11-0699-42	1:60
CD103 PE Cy7	eBioscience	B-Ly7	25-1038-42	1:150
CD8 PE Cy5.5	eBioscience	RPA-T8	45-0088-42	1:300
IFNgamma APC	BD	B27	554702	1:300
TNF PE Cy7	BD	MAb11	557647	1:60
IL2 FITC	BD	5344.111	340448	1:30
Anti-Human CD28/CD49d Purified	BD	L293, L25	347690	1:50
